# Duox is the primary NADPH oxidase responsible for ROS production during adult caudal fin regeneration in zebrafish

**DOI:** 10.1016/j.isci.2023.106147

**Published:** 2023-02-04

**Authors:** Kunal Chopra, Milda Folkmanaitė, Liam Stockdale, Vishali Shathish, Shoko Ishibashi, Rachel Bergin, Jorge Amich, Enrique Amaya

**Affiliations:** 1 Division of Cell Matrix Biology & Regenerative Medicine, School of Biological Sciences, Faculty of Biology, Medicine and Health, University of Manchester, Manchester M13 9PT, UK; 2 Manchester Fungal Infection Group (MFIG), Division of Evolution, Infection, and Genomics, School of Biological Sciences, Faculty of Biology, Medicine and Health, University of Manchester, Manchester M13 9PT, UK; 3 Mycology Reference Laboratory, National Centre for Microbiology, Instituto de Salud Carlos III (ISCIII), Majadahonda 28220 Madrid, Spain

**Keywords:** Cell biology, Ichthyology, Molecular genetics

## Abstract

Sustained elevated levels of reactive oxygen species (ROS) have been shown to be essential for regeneration in many organisms. This has been shown primarily via the use of pharmacological inhibitors targeting the family of NADPH oxidases (NOXes). To identify the specific NOXes involved in ROS production during adult caudal fin regeneration in zebrafish, we generated *nox* mutants for *duox*, *nox5* and *cyba* (a key subunit of NOXes 1–4) and crossed these lines with a transgenic line ubiquitously expressing *HyPer*, which permits the measurement of ROS levels. Homozygous *duox* mutants had the greatest effect on ROS levels and rate of fin regeneration among the single mutants. However, *duox*:*cyba* double mutants showed a greater effect on fin regeneration than the single *duox* mutants, suggesting that Nox1-4 also play a role during regeneration. This work also serendipitously found that ROS levels in amputated adult zebrafish fins oscillate with a circadian rhythm.

## Highlights

• Fin amputation results in elevated ROS that oscillate with the circadian clock
• *duox* mutants have diminished ROS levels following caudal fin amputation
• *duox* mutants have diminished rate of caudal fin regeneration
• *duox/cyba* mutants have a more severe defect in regeneration than *duox* mutants

## Graphical abstract

**Figure:**
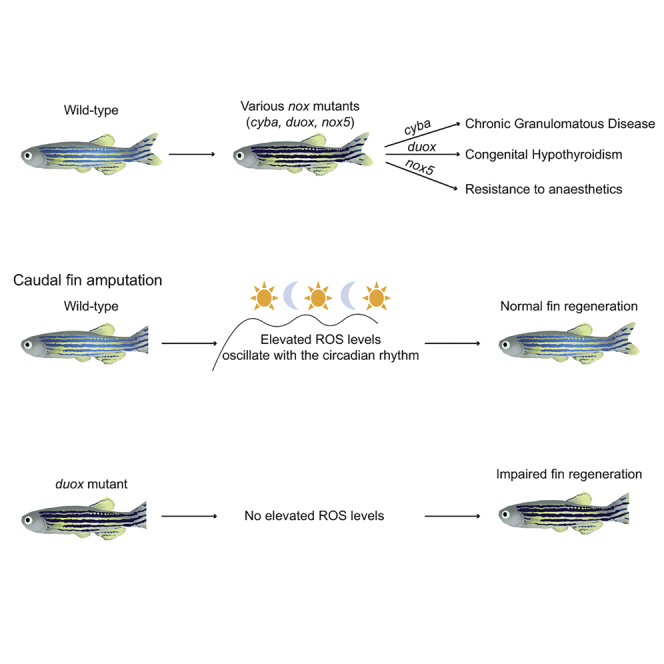

## Introduction

A major aim in the field of regenerative biology is to uncover the mechanisms employed by various species in the animal kingdom, enabling them to regenerate organs and appendages, and in some cases, entire bodies. Deciphering such mechanisms, especially those that are evolutionarily conserved, will provide insight into why some animals have greater regenerative potential than others. Importantly, uncovering those highly conserved mechanisms may lead to novel therapies that could awaken a better regenerative response, clinically, in the sister field of regenerative medicine.

Over the past decade, it has become increasingly apparent that elevated reactive oxygen species (ROS) levels play an essential role during whole-body, appendage, organ, and tissue regeneration across the animal kingdom, including *Hydra*, planarians, *Drosophila*, zebrafish, axolotl, *Xenopus*, reptiles, and mammals.^1^^,^^2^^,^^3^^,^^4^^,^^5^^,^^6^^,^^7^^,^^8^^,^^9^^,^^10^^,^^11^^,^^12^ A key outstanding question is what are the mechanisms that control ROS production following injury and during regeneration? Findings from several model organisms have shown a critical role for Nicotinamide adenine dinucleotide phosphate (NADPH) oxidases (NOXes) in the production of ROS following injury.^5^^,^^7^^,^^8^^,^^9^^,^^10^^,^^11^^,^^13^^,^^14^ The NOXes comprise a family of transmembrane, sometimes multi-component enzymes, whose primary physiological function is the transfer of an electron across biological membranes onto molecular oxygen to produce superoxide. The superoxide ion, which is an ROS, can then be dismutated to other more stable and readily diffusible ROS forms, such as hydrogen peroxide (H_2_O_2_).^15^ Most previous work establishing a critical role for NOXes during ROS production and regeneration has been based on the use of pharmacological inhibitors such as diphenylene iodonium (DPI), apocynin, and VAS2870, which lack specificity and selectivity.^16^^,^^17^ Therefore, to identify the specific NOXes responsible for ROS generation during adult appendage regeneration, we decided to take a genetic approach using zebrafish. All fins of the adult zebrafish are able to regenerate.^18^ We focused on the caudal fin, which is easily accessible and has unlimited regeneration potential.^19^ Here we report the generation and characterization of several mutant alleles of zebrafish, *cyba*, *nox5*, and *duox*. Using homozygous mutants for each of these genes, as well as *cyba*:*duox* double mutants, we investigated the effect of each mutant on ROS production and adult fin regeneration.

## Results

### Molecular characterization of *nox* mutant alleles

**Figure 1:**
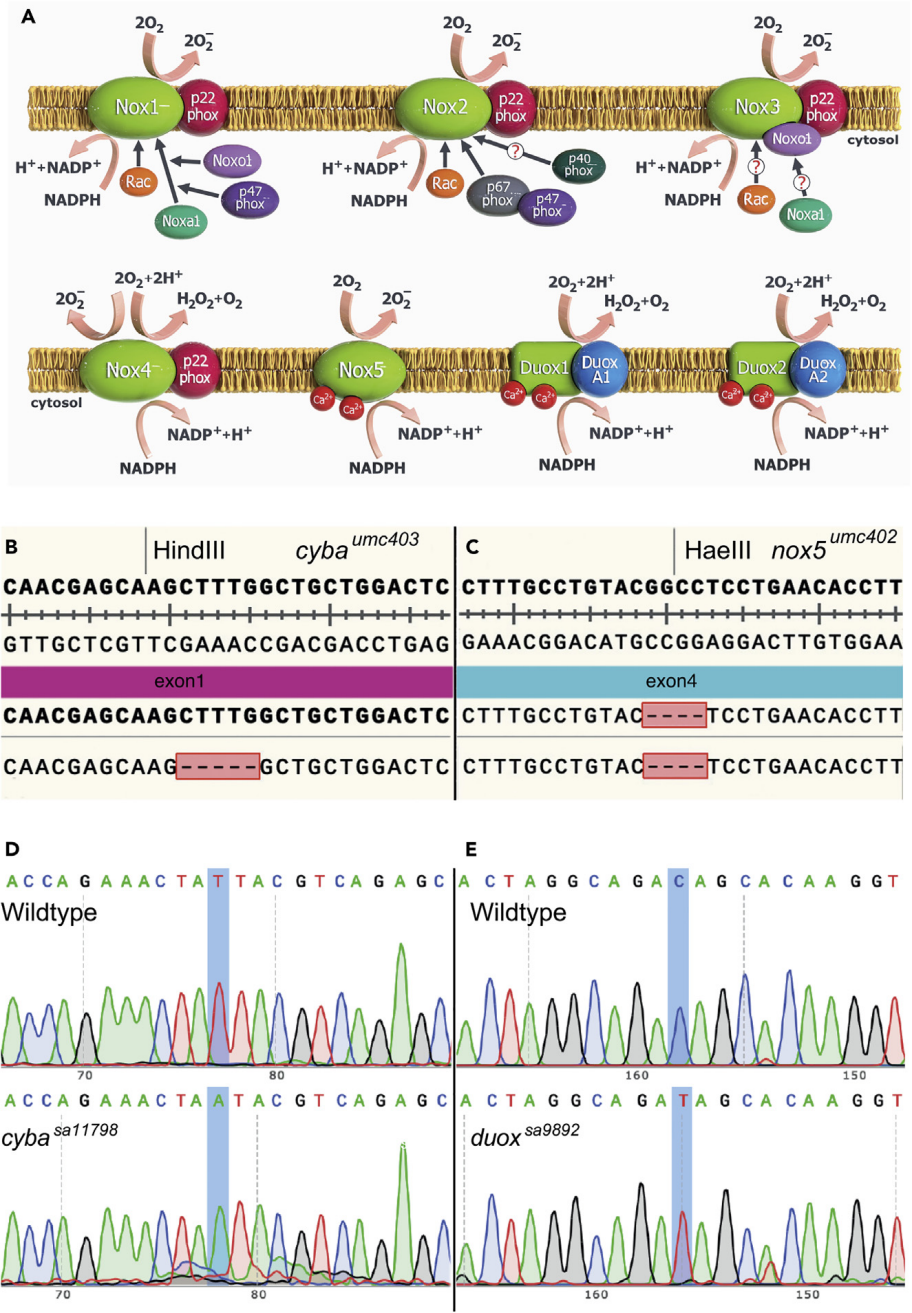
The family of NADPH oxidases (A–E) All NOXes are transmembrane proteins that transport oxygen across biological membranes, reducing oxygen to superoxide, which can then undergo dismutation to generate H_2_O_2_. NOXes are multi-componential proteins, and p22phox (the product of the *cyba* gene) is a common subunit to NOX1-4. Zebrafish lack Nox3 and have a single Duox isoform. Mutants for *cyba* (B) and *nox5* (C) were generated using CRISPR, resulting in a 5bp deletion (*cyba*^*umc403*^) and a 4bp indel (*nox5*^*umc402*^), respectively. The mutations led to the removal of restriction enzyme sites, providing an easy diagnostic method for screening animals. Characterization of *cyba*^*sa11798*^ (D) and *duox*^*sa9892*^ (E) via Sanger sequencing shows the single nucleotide changes T>A and C>T, respectively, in contrast to a WT reference sequence. Panel A courtesy Dr. Kalin Narov, https://kalinnarov.wixsite.com/embryosafari. See also Figures S1 and S2.

The primary aim of this work was to identify the molecular mechanisms responsible for sustained ROS production during zebrafish adult caudal fin regeneration. Previous work has provided evidence that NOXes play an essential role in ROS production and subsequent caudal fin regeneration, but that work was based on the use of NOX inhibitors,^13^ which inhibit all NOXes.^16^ Thus, we chose a genetic approach as a more specific strategy to pinpoint the NOXes. To do so, we sought to obtain or generate mutant alleles for a number of NOXes or their essential subunits (Figure 1A). One such critical subunit, P22^phox^, is encoded by the *CYBA* gene. P22^phox^ plays an essential role in the maturation and structural integrity of NOXes 1–4.^20^ Like the mammalian *CYBA* orthologues, the zebrafish *cyba* genomic locus contains 6 exons and 5 introns.^21^^,^^22^ We also generated a mutant allele for *nox5*, which encodes Nox5, a calcium-regulated single-subunit NOX enzyme (Panday et al., 2015). NOX5 is present in humans but is absent in rodents.^23^ The zebrafish *nox5* has 2 splice variants, both of which are protein coding and feature 16 exons. To try to achieve null mutant alleles for *cyba* and *nox5*, we targeted exon 1 of *cyba* and two sequences in exon 4 (common to both splice variants) for *nox5* using CRISPR. F_0_ adults were crossed to wild-type (WT) animals, and F_1_ *cyba*^*umc403*/+^ and *nox5*^*umc402/+*^ animals were identified via sequencing and subsequently via restriction digest with HindIII and HaeIII, respectively (Figures 1B and 1C). For *cyba*, we found a 5bp deletion, leading to a frameshift mutation. For *nox5*, a 4bp indel resulted from the CRISPR-induced mutagenesis, also resulting in a frameshift mutation. Incrossing F_1_ heterozygotes led to the establishment of stable homozygous mutant lines, hereafter referred to as *cyba*^*umc403*^ and *nox5*^*umc402*^. For *cyba*, we also obtained a nonsense mutant allele, *cyba*^*sa11798*^, from the Zebrafish Mutation Project.^24^ *cyba*^*sa11798*^ is located in exon 4 (Genome assembly: GRCz11), leading to a T>A transversion (Figure 1D) causing a premature stop codon (TAG) after the 87th amino acid of the protein. Homozygous mutants for *cyba* and *nox5* are viable and fertile and display no overt visible phenotypes. The zebrafish genome encodes a single *duox* gene, instead of the two paralogues (*DUOX1* and *DUOX2*) present in tetrapods.^25^ We have previously characterized a *duox* nonsense mutant allele (*duox*^*sa9892*^),^26^ which also arose from the Zebrafish Mutation Project.^24^ The *duox*^*sa9892*^ mutation is located in exon 21 (Genome assembly: GRCz10), resulting in a C>T transition (Figure 1E) and a premature stop codon (TAG) after the 944th amino acid. Homozygous mutants for this allele are viable as adults and display congenital hypothyroidism.^26^

### *cyba and nox5* mutations are linked to unique phenotypes

After generating the *cyba*, *duox*, and *nox5* mutant alleles, we asked whether any of them displayed homozygous mutant phenotypes. We recently showed that *duox*^*sa9892*^ mutants are homozygous viable but display several phenotypes consistent with congenital hypothyroidism, including shorter body length, external goiters, hyperpigmentation, and sterility. These phenotypes could be rescued by T_4_ dosing and could be phenocopied in WT animals via chronic exposure to a goitrogen.^26^

For *cyba*, we took clues from its clinical relevance to chronic granulomatous disease (CGD). In human patients, CGD is often linked to mutations in *CYBA*.^22^ To this end, CGD patients experience recurrent infections, most frequently brought on by *Aspergillus* spp., *Burkholderia cepacia*, *Staphylococcus aureus*, *Serratia marescens*, *Nocardia* spp., and *Salmonella*.^27^^,^^28^^,^^29^^,^^30^ This high sensitivity to bacterial and fungal infections arises from inappropriate or insufficient ROS production in innate immune cells, required to kill engulfed pathogens. Although both zebrafish *cyba* mutant alleles used in this work are homozygous viable and fertile, we wondered whether they might be more susceptible to fungal infections and thus exhibit CGD-like phenotypes. Indeed, the *cyba* mutant line we obtained from the European Zebrafish Resource Center (EZRC) (*cyba*^*sa11798*^*)* had been previously shown to be highly sensitive to fungal infections.^31^ However, we wished to confirm whether the CRISPR-mediated mutant we generated, *cyba*^*umc403*^, was also hypersensitive to fungal infections. Given that invasive infections by filamentous fungi are especially important contributors to morbidity and mortality in CGD patients,^32^^,^^33^^,^^34^ we asked whether *cyba*^*umc403*^ larvae had increased susceptibility to *Aspergillus fumigatus* infections, relative to WT larvae. To achieve this, we took advantage of the large and easily accessible larval yolk sac to inject a suspension of *A*. *fumigatus* conidia into *cyba* mutant and WT larvae. We first asked whether injecting buffer alone could bring about mortality. For this we injected buffer solution in both *cyba* mutant (n = 50) and WT larvae (n = 50) and compared the mortality rates in those versus uninjected cohorts (n = 50 each). The larvae of both genotypes tolerated the injections well, with fewer than 3 deaths each after injection, irrespective of genotype. However, across two independent experiments (Figures S1A and S1B), *cyba*^*umc403*^ animals (n = 25) injected with *A*. *fumigatus* conidia showed a significantly higher mortality rate (40% in A and 86% in B) at 3dpi, compared to WT larvae (n = 17) (17% in A and 27% in B) (log rank test; p value, <0.005) (Figures S1A and S1B). Similar results were obtained following injection of *A*. *fumigatus* conidia into *cyba*^*sa11798*^ larvae (n = 8), with mortality rates at 87% in this instance (Figure S1A). All larvae that developed infections showed similar phenotypes. During the initial stages of infection, the swimming ability of the larvae was significantly affected whereby they could move their pectoral fins but could not achieve productive movement. As the infection advanced, larvae could not remain upright, probably due to effects on the swim bladder (Figures S1C and S1D). Terminal infections were characterized by extensive necroses and feebly moving gill arches and heartbeat. Thus, in agreement with the previous study,^31^ *cyba*^*sa11798*^ animals were very sensitive to *A*. *fumigatus* infection. Similarly, *cyba*^*umc403*^ animals were also ultrasensitive to *A*. *fumigatus* infection. This confirmed that mutations in *cyba* cause high susceptibility to fungal infection in zebrafish larvae, as in human patients with CGD, thus designating both *cyba* mutant alleles as useful models for human CGD.

Based on the existing literature, we had no clues as to the phenotypes that we might expect in homozygous *nox5* mutant fish. No human disease has yet been associated with *NOX5* mutations, and rodents lack a *Nox5* ortholog. While we found *nox5*^*ex4*.*4bp*^ fish to be viable and fertile, we serendipitously uncovered an unusual mild phenotype in these animals after treating them with anesthetics. Intriguingly, most *nox5*^*ex4*.*4bp*^ animals appeared to have prolonged resistance to the commonly used anesthetic, tricaine methanesulfonate (MS-222). To characterize this phenotype further, we individually treated *nox5*^*ex4*.*4bp*^ adults (n = 22) and WT adults (n = 23) with MS-222 (0.04%) in a beaker. We then measured two responses to the anesthetic: 1) righting reflex and 2) cessation of opercular movement. The righting reflex normally serves to resume orientation when the body loses its upright orientation. All WT animals lost the righting reflex in under 30 s following immersion (Figure S2A). After this point, simple tapping of the bench on which the beaker was placed elicited no response from them. In contrast, it took *nox5*^*ex4*.*4bp*^ animals up to 360 s to achieve loss of the righting reflex. Even though they stopped swimming, mutants remained responsive to the tapping, which stimulated jerky, erratic movements. The loss of opercular movement in WT animals took up to 200 s, while in the mutants this took as long as 382 s (Figure S2B). Interestingly, most mutants appeared to lose the righting reflex very close to cessation of opercular movement. We further checked the response of both groups to lidocaine hydrochloride and cold water, both of which have been previously tested as anesthetics in zebrafish.^35^ While neither group achieved a surgical plane of anesthesia with 750 mg/L of lidocaine, loss of the righting reflex was significantly delayed among the *nox5* mutants (Figure S2A). Meanwhile, cold water induced a rapid surgical plane of anesthesia on both groups, with no statistically significant difference in their responses (Figure S2).

Overall, we found that, while all *nox* mutant alleles were homozygous viable into adulthood, each displayed unique phenotypes, suggesting that all mutants were at least hypomorphic, if not null mutant alleles.

### Caudal fin regeneration in zebrafish is not influenced by age

**Figure 2:**
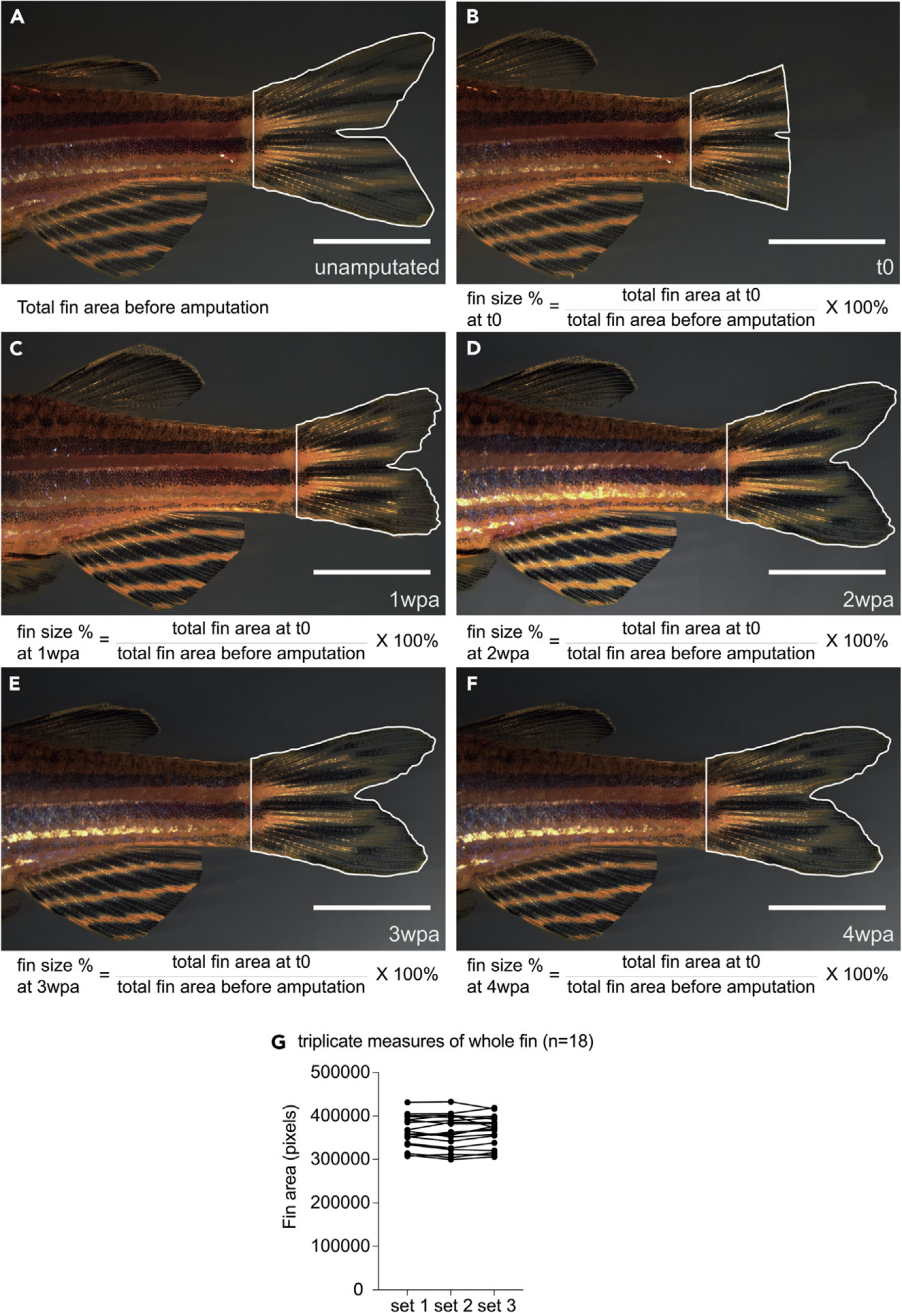
Methodology for quantifying rate of adult fin regeneration (A–F) Amputated adult caudal fins in zebrafish achieve complete, scar-free regeneration by 4 weeks post-amputation (wpa). In all cases, the fin was imaged prior to amputation (A), immediately following amputation (B) and then once weekly at 1wpa (C), 2wpa (D), 3wpa (E), and 4wpa (F). The distinctive stripe and pigment patterns on the body and anal fin enabled identification of individual animals. Fins were amputated midway, and the caudal peduncle was used to demarcate the proximal extent. Scale bar = 5 mm. (G) Repeat (triplicate) measurements of caudal fin areas in 18 adult fish (n = 18). See also Figures S3, S4, Tables S1, and S2.

Given that we would be assessing fin regeneration in animals of different genotypes and ages throughout this study, we performed caudal fin amputations in WT fish of different ages to ascertain whether their age might affect the extent and/or rate of fin regeneration. For this analysis, we amputated approximately 50% of the caudal fin in adult animals from 3 months through 22 months of age and then followed them individually over the four weeks it normally takes for fin regeneration to complete. Given that we were interested in regeneration of the entire fin, as opposed to specific parts of the fins, such as the rays, we first measured the size of the entire caudal fin of individual animals immediately prior to and after amputation. We then measured the caudal fins of each individual once a week for up to four weeks and determined the extent of regeneration as shown in Figures 2A–2F. However, we were cognizant of the potential variability in measuring the fin area, due to the variable extent of fanning out of the fin during repeat measurements over time. To investigate whether this might be an issue, we first performed repeat measurements on 18 individual animals prior to amputation (where each individual was anesthetized, imaged, and allowed to recover for a total of three times). We found that, if the animals are allowed to relax fully following each anesthesia event before imaging, the fin area measurements were very similar over the triplicate measurements (Figure 2G). The variability across the triplicate measurements per animal was generally between 1% and 4%, and there was no statistical difference between the triplicate measurements within individuals (Table S1). In contrast, there was a significant difference in the fin areas between individuals (Table S2), which is unsurprising given that zebrafish display markedly different growth rates even from the same clutch.^36^ Thus, given the greater variability between animals than within repeat measurements within individuals, we decided to assess regeneration by following individuals, rather than assessing regeneration by following the population, as is routinely done in the field.^37^^,^^38^^,^^39^ Using this approach, we measured the extent of regeneration in individual animals of different ages and we found that adult zebrafish younger than six months of age generally overshot the initial size of their fins over the four weeks after amputation (i.e., regenerated to over 100% of their initial fin size) (Figures S3A–S3C). However, caudal fins in fish six months and older generally only grew back to their original fin size (Figures S3D–S3I). From this we can conclude that caudal fin regeneration in young adult fish results from a combination of fin regeneration and growth, while in fish older than 6 months regeneration of the fin is not confounded significantly by fin growth as long as regeneration is not followed over more than four weeks post-amputation. We thus decided to concentrate the remainder of our tail fin amputation experiments on fish six months or older.

Next, we were interested in addressing whether the rate of fin regeneration is affected by the age of the fish. To answer this question, we plotted the slope of regeneration over the first, second, third, and fourth week of regeneration as a function of age (Figures S4A–S4E). We found that, overall, caudal fin regeneration is the fastest during the first two weeks of regeneration (week 0–1 and week 1–2) and then slows down during the third (week 2–3) and fourth week (week 3–4) (Figure S4F). Thus, the rate of caudal fin regeneration is biphasic, with similar rates between the first and second weeks and between the third and fourth weeks, with the latter two statistically slower than the first two weeks (Figure S4F). Furthermore, we found that animals between six and eight months of age regenerated at the same rate over the four-week period, while older animals exhibited statistically different regeneration rates relative to animals of other ages during at least one time point (Figures S4A–S4E). These results provided us with a good “working age range” of animals, where neither growth nor rates of regeneration were significantly affected by the age of the animals, and thus we concentrated subsequent analyses on animals between six and eight months of age.

### *cyba* and *duox* mutations affect caudal fin regeneration

**Figure 3:**
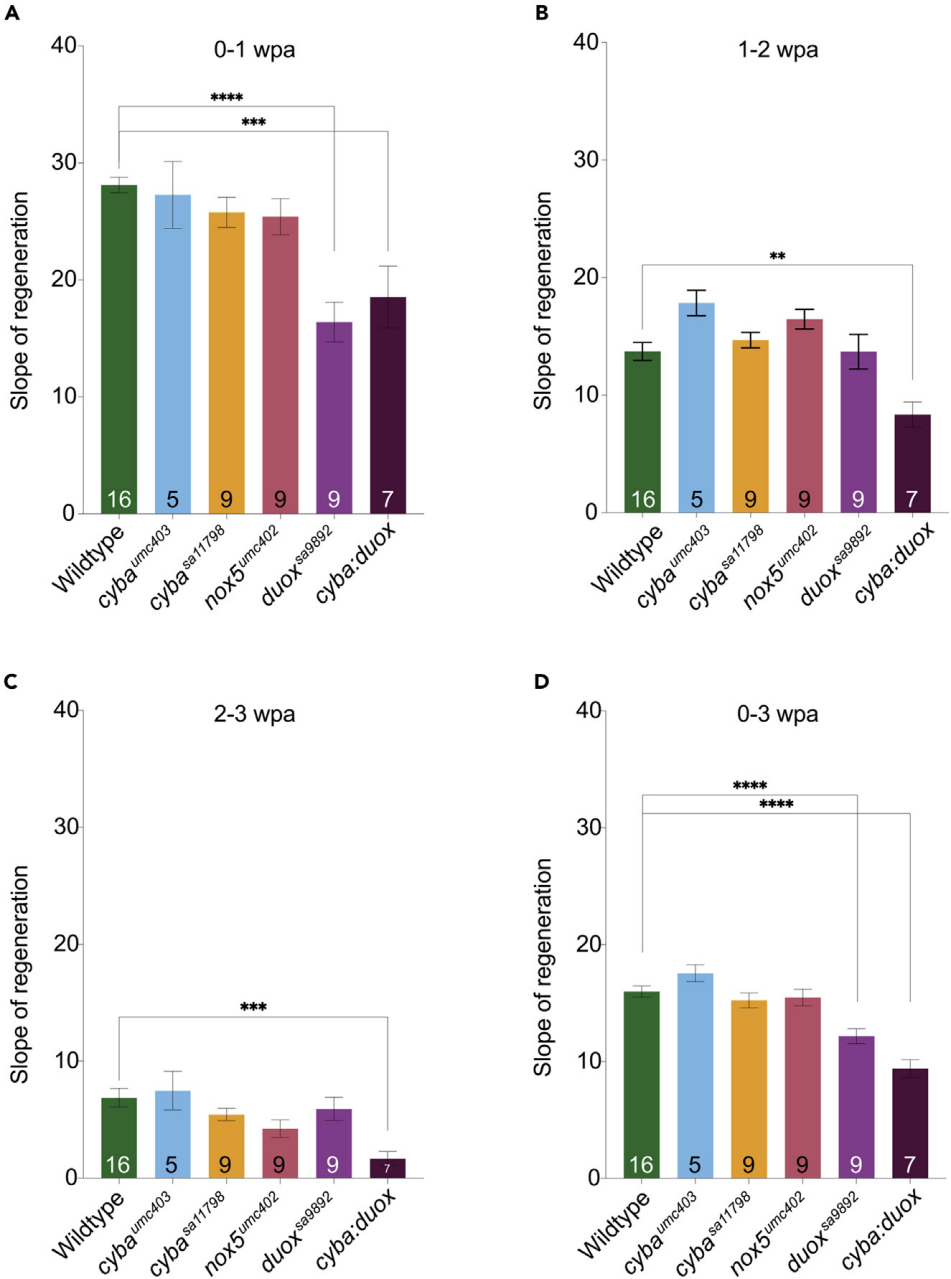
*duox* mutants regenerate the caudal fin slower than WTs (A–C) Significant differences in regeneration were observed between WT and *duox* mutants during 0-1wpa (A) and 1-2wpa (B). By 2-3wpa (C) these differences were resolved, with only the double mutant *cyba*:*duox* continuing to significantly trail behind. (D) An overall view across 0-3wpa highlights how *duox*^*sa9892*^ and *cyba*:*duox* animals are significantly slower than every other group. Asterisks denote statistically significant differences (Bonferroni’s multiple comparisons test, ∗p < 0.5, ∗∗p < 0.01, ∗∗∗∗p < 0.0001). WT (n = 16); *cyba*^*umc403*^ (n = 5); *cyba*^*sa11798*^ (n = 9); *nox5*^*umc402*^ (n = 9); *duox*^*sa9892*^ (n = 9); *cyba*:*duox* (n = 7). See also Figures S5–S8.

Having identified regeneration trends in WT animals, we proceeded to investigate the rate of regeneration in the various *nox* mutants, namely *cyba*^*umc403*^ (n = 5), *cyba*^*sa11798*^ (n = 9), *duox*^*sa9892*^ (n = 15), *nox5*^*umc402*^ (n = 9), and *cyba*^*sa11798*^:*duox*^*sa9892*^ double mutants (n = 7) (hereafter referred to as *cyba*:*duox*), along with their WT siblings (n = 16) (Figures 3 and S5). All caudal fin amputations were performed on animals between six and eight months of age. In addition, we took care to include only animals that had similar amounts of the caudal fins amputated (between 40% of 55% of the original fin size) for subsequent analyses as evidence from others has shown that the rate of fin regeneration may be affected by the amount of fin that is amputated.^39^^,^^40^^,^^41^ Once all of these factors were taken into consideration, we found that, out of the various *nox* mutants, only *duox*^*sa9892*^ and *cyba*:*duox* animals displayed a significant effect on the rate or extent of adult caudal fin regeneration (Figure 3). More specifically, we found that *duox*^*sa9892*^ animals had a significantly slower rate of regeneration when compared to WTs during the first week following amputation (Figure 3A). This delayed rate of regeneration was further amplified in the *cyba*:*duox* double mutants, where the slower rate of regeneration continued into the third week (Figures 3B and 3C). It is important to point out that, while regeneration in these two mutant strains was not entirely inhibited, these mutants exhibited a significant slowing in the rate of regeneration over the 3 weeks that they were allowed to regenerate. In particular, the overall size of the regenerating fins in *duox*^*sa9892*^ and *cyba*:*duox* animals remained significantly smaller than that in the WT cohorts (Figure S6). Thus, the overall defect in regeneration appeared to be in the rate of regeneration, more so than on overall regeneration per se. To identify the predicted timescales expected for the completion of regeneration, i.e., the time likely to take for the fins to reach the unamputated size, we used row statistics with slopes derived from simple linear regression (Figures S7 and S8). This revealed that WTs, *cyba*, and *nox5* mutants would complete regeneration in under 5 weeks post amputation (wpa) (Figures S8A–S8D), while *duox*^*sa9892*^ animals would not be expected to reach their pre-amputation sizes until 7.3wpa (Figure S8E), and *cyba*:*duox* animals would not achieve their pre-amputation sizes until 11.2wpa (Figure S8F). Thus the *duox*^*sa9892*^ and *cyba*:*duox* mutants displayed a 2–3 times slower rate in tail fin regeneration relative to WT animals or the other *nox* single mutants assessed in this study.

### The effect of hypothyroidism on the rate of fin regeneration

**Figure 4:**
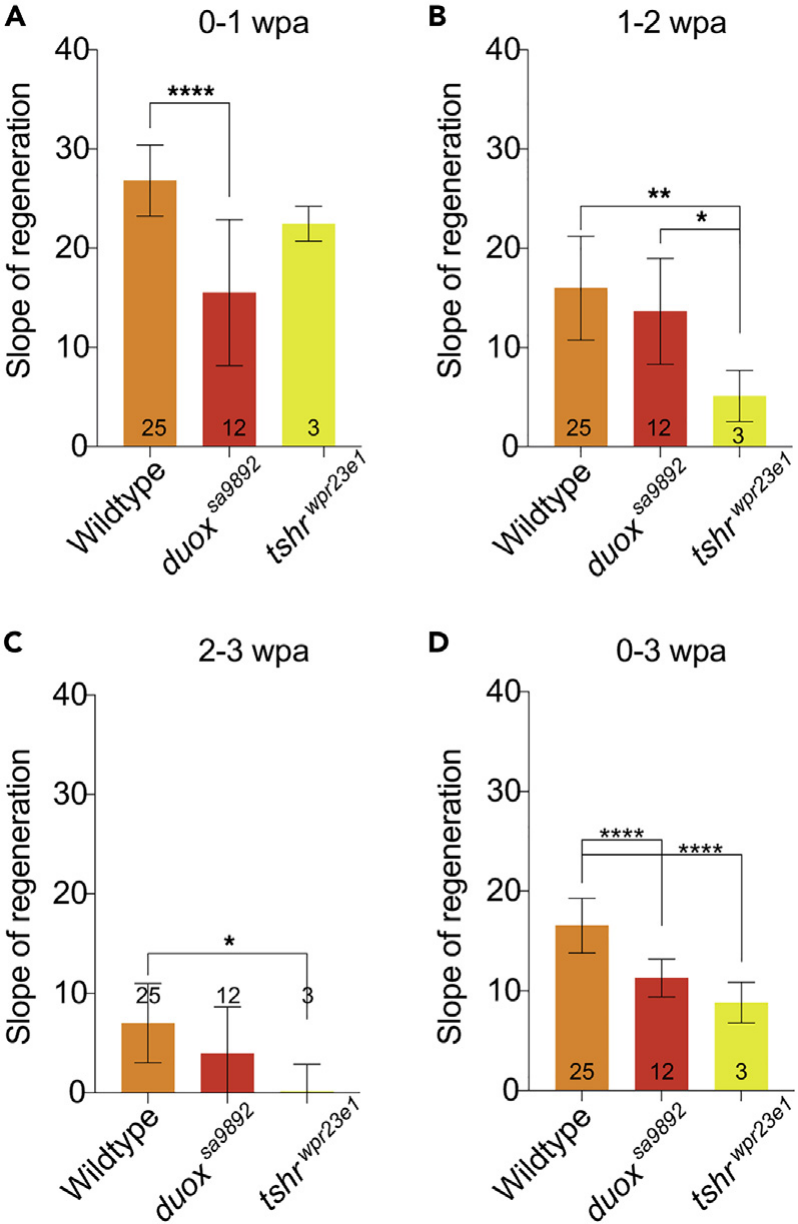
Thyroid hormone deficiency slows down caudal fin regeneration but only during the late phases of regeneration (A–C) The impact of *duox*^*sa9892*^, but not *tshr^wpr23e1^*, on the rate of regeneration is significant during 0-1wpa. By 2wpa (B) the effect of *tshr^wpr23e1^* becomes significant, and this effect continues into 2-3wpa (C). (D) An overall view across 0-3wpa indicates *duox*^*sa9892*^ and *tshr^wpr23e1^* animals are significantly slower than WTs during regeneration. Asterisks denote statistically significant differences (Bonferroni’s multiple comparisons test, ∗p < 0.5, ∗∗p < 0.01, ∗∗∗∗p < 0.0001). WT (n = 25); *duox*^*sa9892*^ (n = 12); *tshr^wpr23e1^* (n = 3). See also Figure S9.

Recently, we reported that *duox^sa9892^* mutant zebrafish exhibit congenital hypothyroidism.^26^ We thus wondered whether the reduced rate of regeneration among the *duox*^*sa9892*^ animals could be attributed to their hypothyroidism. We therefore investigated fin regeneration using another zebrafish model of congenital hypothyroidism, namely the *tshr^wpr23e1^* mutant (original name *manet^wpr23e1^)*.^42^ This mutant harbors a nonsense mutation in exon 2 in the *tshr* gene, which encodes the thyroid-stimulating hormone (TSH) receptor. We have previously reported phenotypic concordance between *tshr^wpr23e1^* and *duox* mutants.^26^ Conveniently, the *tshr^wpr23e1^* allele destroys an HpyCH4V restriction site, providing an easy diagnostic method for genotyping. The effect of the *tshr* mutation on body length had not been previously reported, so we measured the body length of WT, *tshr^wpr23e1^*^*/+*^, and *tshr^wpr23e1^* siblings at 3 months of age. This assessment showed a similar growth retardation phenotype that we previously reported in *duox*^*sa9892*^ mutants^26^ (Figure S9). We proceeded to amputate the caudal fin in WT, *tshr^wpr23e1^*, and *duox*^*sa9892*^ fish. In the first wpa, a significantly slower rate of regeneration was observed in *duox*^*sa9892*^, when compared to WT animals or *tshr^wpr23e1^* fish (Figure 4A). However, during the second and third weeks of regeneration, the *duox*^*sa9892*^ and WT fish had no significant difference in their rates of regeneration (Figures 4B and 4C). In contrast, the *tshr^wpr23e1^* fish regenerated at the same rate as WT fish during the first week (Figure 4A), but then their regeneration rate slowed down relative to both WT and *duox*^*sa9892*^ fish during the second and third weeks of regeneration (Figures 4B and 4C). In summary, both *duox*^*sa9892*^ and *tshr^wpr23e1^ animals* displayed delayed rates of fin regeneration over the three-week period (Figure 4D). However, the *duox*^*sa9892*^ animals displayed the greatest impact on fin regeneration during the initial phases of regeneration, while hypothyroidism alone decelerates it only during the later stages, suggesting that the effect of *duox* on regeneration is not solely due to their hypothyroidism.

### *ubb*:*HyPer* is an effective reporter of amputation-induced ROS production

**Figure 5:**
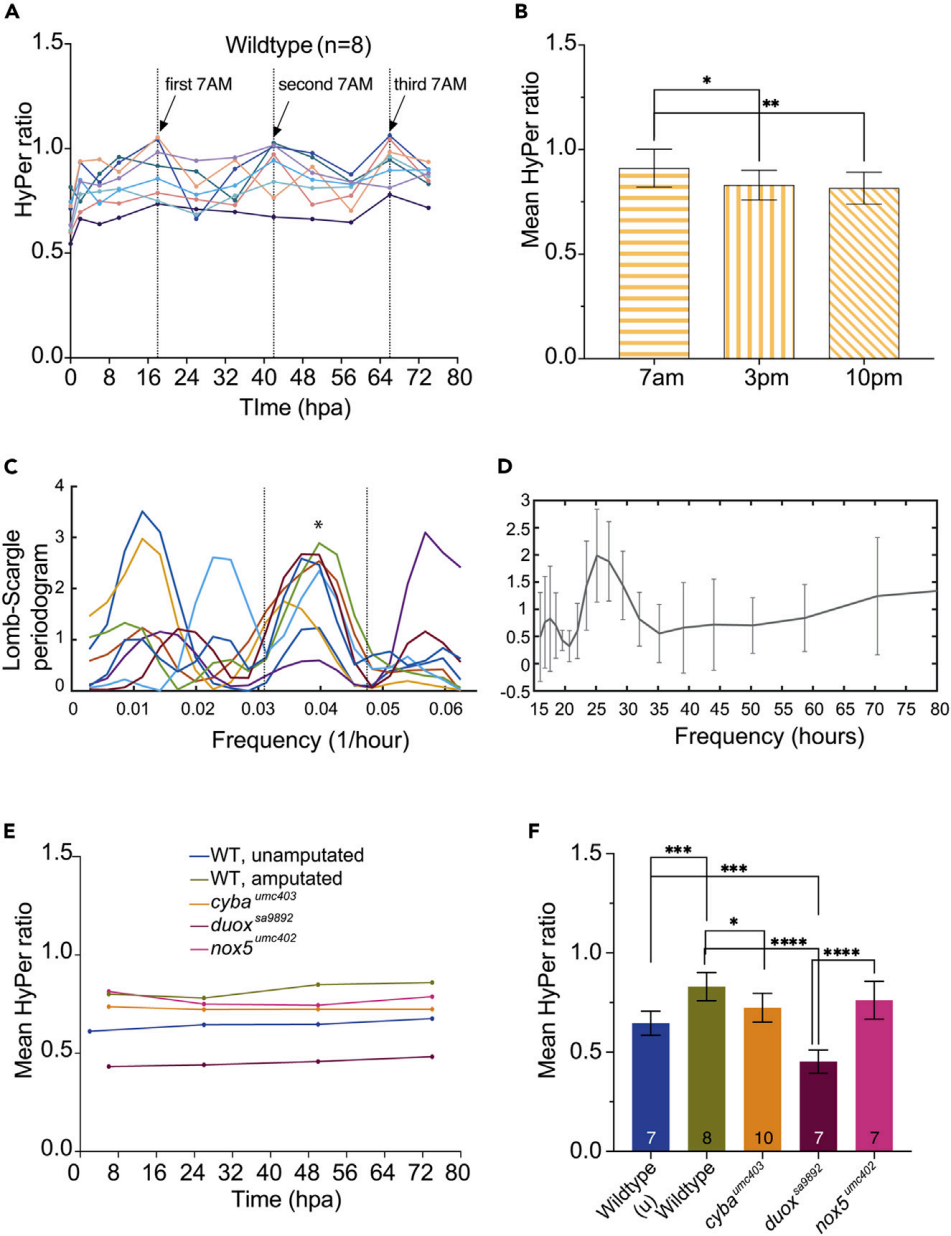
HyPer ratios in WT animals and *nox* mutants WT animals and *nox* mutants were generated in a *Tg(ubb*:*HyPer)umc400* transgenic background, which permits *in vivo* assessment of ROS levels. (A) ROS Hyper ratios in individual WT animals (n = 8) observed at three time points during the day over three days post-amputation reveal an oscillatory pattern of ROS levels. (B) ROS levels over three consecutive days post-amputation were significantly higher in the mornings (7a.m.) than in the afternoons (3 p.m.) or nights (10p.m.). (C and D) Long-Scargle periodogram reveals that the ROS oscillations follow a daytime-dependent trend, with a periodicity of around 0.4 (∗ on graph), indicative of a ∼24 h circadian cycle (D). (E) Graph of ROS levels measured at the same time of day (3p.m.) in WT (unamputated), WT (amputated), *cyba*^*umc403*^, *duox*^*sa9892*^, and *nox5*^*umc402*^ animals. (F) Graph of mean Hyper ratios measured over three consecutive days at 3p.m. reveals significantly attenuated post-amputation ROS levels among the *duox*^*sa9892*^ mutants relative to the WT amputated fins and the other *nox* mutants. Indeed, ROS levels in the *duox*^*sa9892*^ fins are significantly lower than even those found in unamputated WT fins. ROS levels in the amputated *cyba* mutant fins are significantly lower than those present in WT amputated fins but not as low as in the *duox*^*sa9892*^ fins. ROS levels in the amputated *nox5*^*umc402*^ mutant fins are not significantly different from those present in amputated WT fins. (Bonferroni’s multiple comparisons test, ∗p < 0.5, ∗∗p < 0.01, ∗∗∗∗p < 0.0001). For A-D, WT (n = 8). For E-F, WT unamputated (n = 7); WT amputated (n = 8); *cyba*^*umc403*^ (n = 10); *duox*^*sa9892*^ (n = 7); *nox5*^*umc402*^ (n = 7). See also Figures S10 and S11.

Given that the slower rate of regeneration during the first week in the *duox* mutants is not linked to their hypothyroidism, we then asked whether it might be due to a diminished production of ROS in the mutants during the early phase of regeneration. To test this hypothesis, we generated a transgenic line that expresses the H_2_O_2_-specific ROS sensor, HyPer,^43^ under the control of the ubiquitous *ubb* promoter (*ubb*:*HyPer*), referred to as *Tg(ubb*:*HyPer)umc400*. We first measured ROS levels in unamputated caudal fins of individual control *Tg(ubb*:*HyPer)umc400* adults (n = 7) (Figures S10A and S10B), following each animal over the course of four days. Individual animals were identified based on their scale morphology, which is very distinctive under fluorescence. Using a set of repeated time points during the day over four days, we aimed to identify the “baseline” H_2_O_2_ levels in adult fins. While basal H_2_O_2_ levels in uninjured fins were relatively stable throughout the four-day period (Figure S7B), ROS levels rose significantly above this baseline level following fin amputation (Figures S10C and S10D). For consistency, we performed all fin amputations around midday. Observations were recorded at the same time intervals as those used for the unamputated controls, with additional time points at 2, 6, and 10 hours post amputation (hpa) (Figure S10D). Unlike the unamputated cohort, these were followed for two weeks. By 2hpa (around 3 p.m.), ROS levels showed a significant increase (paired t-test, p = 0.0009) above the unamputated level measured at the same time. At 6hpa and 10hpa, the increase in ROS levels was sustained, but thereafter the levels appeared to oscillate each day with the highest levels in the mornings (between 7 a.m. and 8 a.m.) and the lowest levels in the afternoons (between 3 p.m. and 4 p.m.) (Figures 5A and S10D). This rise and fall in ROS levels was recorded over three consecutive days. After three days, the frequency of recordings was reduced to once daily, between 3 p.m. and 4 p.m. After the sixth day, the frequency of imaging sessions was further reduced to once every two days, between 3 p.m. and 4 p.m., at which point ROS levels were still higher than the baseline, pre-amputation levels (Figure S10D). The final recording was made mid-afternoon (between 3 p.m. and 4 p.m.) two weeks after amputation. At this point the ROS levels had finally returned to pre-amputation baseline levels (Figure S10D).

We then asked whether the ROS levels measured over consecutive days taken at the same time of the day differed significantly from ROS levels measured at other times of the day. This analysis showed that ROS levels measured at 7 a.m. were significantly higher than those measured at either 3 p.m. or 10 p.m., over the three consecutive days (Figure 5B). To confirm the veracity of these apparent oscillations, we then performed a Fourier transform power spectrum analysis on the changing ROS levels of all the amputated fins over the three days of measurements (Figures 5C and 5D). This analysis showed a coordinated high periodicity peak in ROS levels in all fish, at a frequency of 0.04 1/h (equating to a periodicity of ∼24 h) (Figures 5C and 5D). Thus, we found that adult fin amputation led to a sustained increase in ROS levels over the first two weeks of regeneration and that ROS levels in the amputated fins oscillate with a circadian rhythm.

### Post-amputation ROS production is affected in the *nox* mutants

Having determined the post-amputation sustained increase in ROS levels in WT fish (Figures S10D and S11A), we next crossed or generated genetically altered lines for all the *nox* mutants in the *Tg(ubb*:*HyPer)umc400* background. We then assessed whether post-amputation ROS levels were altered in these mutants. We found that the *cyba*^*umc403*^ animals (n = 10) displayed an increase in ROS levels post-amputation (t-test, p = 0.0004) (Figure S11B). Interestingly, the *cyba* mutants returned to pre-amputation ROS levels by 1wpa (168hpa) (Figure S11B), in contrast to the WT animals, where pre-amputation ROS levels were not seen until 2wpa (Figure S10D). Also, the oscillations in ROS levels during the day exhibited peaks of smaller amplitudes and had a lower H_2_O_2_ increase overall when compared to WT fish. Furthermore, H_2_O_2_ levels in *cyba* mutants were similar to those found in WT fish at 2hpa but had lower values at later time points.

In contrast, *duox*^*sa9892*^ animals (n = 7) (Figure S11C) showed low ROS levels post-amputation throughout the week-long course of observations. The post-amputation levels were significantly lower at all time points taken, and there were no distinctive peaks associated with the 7 a.m. time points. Even more interestingly, ROS levels were lower than those present in WT unamputated controls (Figures 5D and S11A and S11C). Finally, the *nox5*^*ex4*.*4bp*^ animals (n = 8) displayed a significant increase in ROS levels at 2hpa (3 p.m.) (t-test, p = 0.04), which peaked at 7 a.m. (18hpa) the next morning (t-test, p = 0.036), and rapidly “baselined”, showing no further significant increase thereafter (Figure S11D). In order to better compare the HyPer ratios between the different genetic backgrounds we superimposed the ratios obtained for all the genetic strains in Figure S11E. Given that we previously found significant differences between the HyPer ratios in WT animals post-amputation depending on the time of the day, we also compared the HyPer ratios across all the *nox* homozygous mutant strains observed at the same time of day post-amputation (i.e. 3 p.m.) over the first three days post-amputation alongside the ratios found in unamputated tail fins (Figure 5D). This analysis confirmed that *duox*^*sa9892*^ animals exhibited the most profound effect on ROS levels following amputation, where no increase was observed in these animals. Furthermore, it confirmed that *duox*^*sa9892*^ tail fins have significantly lower ROS levels even in the pre-amputation state, when compared to all other genotypes. The other *nox* mutants had a much milder effect on post-amputation ROS levels, and unlike the *duox* mutants, none of the other *nox* mutants affected the initial increase in ROS levels caused soon after fin amputation (Figure 5D).

Overall, these results pinpoint Duox as the primary Nox responsible for ROS production following injury and during regeneration, with the other NOXes also potentially involved in ROS production but to a much lesser extent. In particular, *cyba* mutants returned to pre-amputation ROS levels after 1wpa, in contrast to WT animals, where elevated ROS levels were sustained over the first 2wpa. This might help explain why the *cyba*:*duox* double mutants have a stronger effect on regenerative capacity than the *duox* single mutants during the later phases of tail fin regeneration.

## Discussion

In this work, we set out to uncover the mechanisms of ROS production following zebrafish caudal fin amputation, with particular emphasis on the NOX family of enzymes. While an essential role for ROS production, mediated primarily by NOXes, in whole organism and appendage regeneration is well documented in the literature, most studies addressing this role were primarily based on the use of chemical inhibitors targeting unspecifically the entire family of NOXes.^2^^,^^5^^,^^7^^,^^8^^,^^9^^,^^10^^,^^11^ In an attempt to pinpoint which NOXes are primarily responsible for ROS production following injury and subsequent regeneration in adult zebrafish, we employed a genetic approach in this study. An initial concern arising from this was the potential complication of genetic redundancy present in teleost fish. Following the divergence of the ray-finned and lobe-finned fishes, teleosts underwent an additional whole genome duplication event in the common ancestor of zebrafish and the other 22,000 ray-finned species.^44^ Despite this concern we found that the zebrafish genome does not harbor additional duplicated paralogues for *cyba*, *duox*, or *nox5* when compared to other non-teleost vertebrates. In fact, we and others have found that zebrafish has only a single *duox* gene, as opposed to most other vertebrates, including mice and humans, which have two *duox* paralogues (*DUOX1* and *DUOX2*).^25^^,^^26^ It should be noted at this point that the zebrafish genome currently has a gene mis-annotated as *duox2* (ZBD GENE 111007-1). This gene is not a paralog of *duox* but instead is the zebrafish paralog of *duoxa*, which encodes a Duox maturation factor.^45^ Duox maturation factors are ER proteins that are essential partners of the *DUOXes*, helping prevent their retention in the ER.^15^ In conclusion, the zebrafish genome contains only a single *duox* paralog, as opposed to most other vertebrates, which harbor two paralogues. Therefore, our work suffered less complications arising from excessive genetic redundancy than it would have if it were carried out in most other vertebrate model systems.

Another potential complication could have arisen if homozygous mutants for any of the genes used in this work had been non-viable as adults. Fortunately, this was not the case. Although viable, we found that homozygous mutants for all three genes did display phenotypes, ranging from hypothyroidism and sterility (*duox*) to enhanced susceptibility to fungal infections (*cyba*) and increased resistance to some anesthetics (*nox5*). Unsurprisingly, the phenotypes associated with *duox* and *cyba* mutations in zebrafish were consistent with mutations in the human orthologs of these genes, with *DUOX2* and *CYBA*, leading to congenital hypothyroidism and chronic granulomatous disease, respectively.^22^^,^^46^ Thus, mutations in the zebrafish orthologs of these two genes provide useful models for these human genetic diseases. In contrast, there are currently no human genetic diseases associated with mutations in *NOX5*, and so we had no prior knowledge of what phenotypes might be associated with mutations in zebrafish *nox5*. In addition, no information can be inferred from mouse knockout studies as rodents lack a *Nox5* orthologue. Serendipitously, we noticed *nox5*^*umc402*^ fish were particularly resistant to some anesthetics, including tricaine and lidocaine. However, we do not currently know the reason behind this. In summary, all the *nox* mutants we used in this work were homozygous viable and each presented with identifiable phenotypes, when appropriately challenged.

The influence of advancing age on regenerative decline is known among adult fish.^47^^,^^48^ Given that we would be using adults, we wanted to identify an age range where the rate of fin regeneration would not be affected by the age of the fish being used. We therefore performed fin regeneration assays, including rate of regeneration, in an array of age groups and found that young adults, from six to eight months of age, showed no significant difference in their rates of fin regeneration. We then set out to assess how each mutation, singly or in combination, affected ROS production following fin amputation and during fin regeneration. Our results showed that, among the single mutants, the *duox* mutants were the most striking, resulting in a significantly lower ROS levels and slower rate of regeneration, especially over the first two weeks following amputation. Furthermore, combinatorial mutants of *duox* and *cyba* displayed an even stronger effect on the rate of fin regeneration, extending into the third week following amputation, suggesting that Duox functions in combination with other NOXes during the later stages of adult fin regeneration in zebrafish. Further support that Duox works in combination with other NOXes to generate the full complement of ROS activated during caudal fin regeneration can be gleaned by the observation that addition of the chemical inhibitors targeting all NOXes resulted in a more pronounced diminution of ROS levels and a stronger effect on caudal fin regeneration^13^ than what we observed in this study in *duox* or *duox*:*cyba* mutant animals. Previously, genetic approaches have also identified a primary role for the Duox maturation factor in ROS generation during *Drosophila* imaginal wing disc regeneration,^4^ suggesting this role for Duox in regeneration may be an ancient, conserved function. This is likely due to the fact that Duox is regulated by calcium and a rise in intracellular calcium is a conserved rapid response following injury.^14^^,^^49^^,^^50^ Furthermore, previous studies have implicated NOX2 during axonal regeneration in mammals.^12^ Intriguingly, Nox2 is one of the multi-component NOXes, whose activity is dependent on Cyba, which encodes p22^*phox*^, also sometimes referred to as the α subunit of Nox2.^20^ Thus, our *cyba* zebrafish mutants would be expected to have impaired Nox2 activity. While we did not find an overt impairment in the rate of caudal fin regeneration in the *cyba* mutants, we found that these animals failed to sustain elevated levels of ROS beyond the first week post-amputation, unlike the WT controls. While this difference in ROS levels was not sufficient to result in a change in the rate of regeneration in the *cyba* alone mutants, we did find that the *duox*:*cyba* double mutants had a more pronounced effect on the rate of caudal fin regeneration, especially after the second week of regeneration, than *duox* alone mutants. Given that our work was primarily interested in overt appendage regeneration, we did not investigate whether the *cyba* mutants exhibited defects in axonal regeneration during caudal fin regeneration, but this would be an interesting question to explore in the future.

While the focal point of this work was the genetic dissection of the role of NOXes in ROS production during adult fin regeneration, our work uncovered some unexpected findings. One was that ROS levels not only rise after fin amputation but also oscillate with a circadian rhythm in the days following injury, with the highest levels found in the mornings (around 7 p.m.) and the lowest levels in the mid-afternoons (around 3 p.m.). Discovering this was important as it called for the need to measure ROS levels at the same time daily, for a more accurate assessment of post-amputation ROS levels in the WT versus mutant fish, without being complicated by the circadian-associated oscillatory changes at different times of the day. However, this finding raises several questions. For example, what is responsible for the circadian-associated oscillations in injury-induced ROS levels? One possible link might be the well-appreciated oscillations in metabolism that are known to be linked to the circadian clock.^51^ We noted that these oscillations were mostly apparent following injury, which might be linked to the injury-induced changes in metabolism that have previously been highlighted in both *Xenopus* tail regeneration and zebrafish appendage and heart regeneration.^52^^,^^53^^,^^54^^,^^55^^,^^56^ It is thus possible that the oscillating levels of ROS are direct or indirect outputs of the metabolic state in the fin following injury. It is also notable that wound healing in mice and humans is affected by the time of day that the injuries are incurred or if the circadian clock is disturbed.^57^^,^^58^^,^^59^^,^^60^^,^^61^ It will be interesting to investigate whether the time of fin amputation affects wound healing or the speed or quality of regeneration in zebrafish, and if so, whether these effects are linked to the dynamics or peak levels of ROS production following injury.

### Limitations of the study

In this study, we used single and compound Nox mutants to assess the specific and combinatorial roles for different Nox during caudal fin regeneration. However, given that there are seven NOXes, it was too onerous a task to generate mutations targeting each of the seven NOXes, individually. Therefore, we chose to target the multi-subunit sub-family of NOXes, namely Nox1-4, by targeting their shared subunit, namely *cyba*. Thus, this study is unable to assess the involvement of Nox1 vs Nox2 vs Nox3 vs Nox4 during caudal fin regeneration but instead used an approach that would affect all of these at the same time. However, given that the *cyba* mutants had no discernible effect on caudal fin regeneration on their own and an effect is only found when these are combined with *duox* homozygous mutants, we believe that not having targeted each Nox separately is less of a concern. Secondly, it is not known whether the mutant alleles used in this study are null mutants or hypomorphs, and this would impact on the interpretation of the study. Furthermore, it is not known whether compensatory mechanisms, as described by El-Brolosy et al,^62^ may have led to less severe consequences of the mutations on ROS production and/or fin regeneration, given that all of these mutant alleles include either frameshift mutations or nonsense mutations and thus would be expected to stimulate nonsense-mediated mRNA decay. Finally, all the cells in the animals used in this study were mutant, and thus, it is difficult to know whether some of the effects on regeneration might have been caused, at least in part, by systemic effects of the mutations on fin regeneration, rather than local requirements for those genes on these processes; this is especially the case with the *duox* mutants, which we know can have systemic effects, including hypothyroidism. Another potential limitation in the interpretations of this study is that the measurements of ROS levels were performed using a genetic sensor, HyPer, which is sensitive to changes in ROS levels but is also sensitive to changes in pH levels. We could determine whether the changes in HyPer ratio are caused by changes in pH levels. For this we would have to establish a transgenic line that expresses a pH-sensitive, but ROS-insensitive, variant of HyPer, called SypHer,^63^ and cross this new line with all the *nox* mutants used in this work, finally followed by incrossing these into homozygosity. However, such a task would be very time consuming and laborious. Thus, we cannot entirely exclude the possibility that some of the changes in HyPer ratio we have documented are not solely due to changes in ROS levels or pH levels or a combination of both. However, we note that we previously addressed this issue during *Xenopus* tadpole tail regeneration, where we were able to show that tadpole tail regeneration is not associated with significant changes in pH levels, and thus changes in HyPer ratios during tadpole tail regeneration were entirely due to changes in ROS levels.^5^

## STAR★Methods

### Key resources table

**Table:** 

REAGENT or RESOURCE	SOURCE	IDENTIFIER
**Chemicals, peptides, and recombinant proteins**		

ExTaq DNA Polymerase	Takara	RR001A
Proteinase K	New England Biolabs®	P8107S
Cas9-NLS protein	New England Biolabs®	M0646T

**Experimental models: Organisms/strains**		

*cyba*^*sa11798*^	European Zebrafish Resource Centre	*ZFIN ID*: *cyba*^*sa11798*^
*duox*^*sa9892*^	European Zebrafish Resource Centre	*ZFIN ID*: *duox*^*sa9892*^
*tshr*^*wpr23e1*^	David Parichy Laboratory	*ZFIN ID*: *tshr*^*wpr23e1*^
Tg(ubb:HyPer)umc400	This publication	*ZFIN ID*: Tg(ubb:HyPer)umc400
*nox5*^*umc402*^	This publication	ZFIN ID: *nox5*^*umc402*^
*cyba*^*umc403*^	This publication	ZFIN ID: *cyba*^*umc403*^

**Oligonucleotides**		

*cyba ex1*.*5bp* F: 5’-AGTTTATTTGCCAGTGACAGCA-3’	Sigma-Aldrich®	Custom
*cyba ex1*.*5bp* R: 5’-CTCAAGCAGCCTACCAAACC-3’	Sigma-Aldrich®	Custom
*cyba sa11798* F: 5’- CTGCTGGTGTGTTTGTGTGT -3’	Sigma-Aldrich®	Custom
*cyba sa11798* R: 3’- AAGGGACATGCTTGACAAGG -5’	Sigma-Aldrich®	Custom
*duox sa9892* F: 5’-ACGAGGTACACAACTCAAGCTG-3’	Sigma-Aldrich®	Custom
*duox sa9892* R: 5’-GACGTTCAAAGCGAAACCTGAC-3’	Sigma-Aldrich®	Custom
*duox sa9892* seq: 5’-CTTGGTCTGCCTTTGACGAAGT-3’	Sigma-Aldrich®	Custom
*manet* F: 5’-TGCAAATTTCGATAAATTGTAATAA-3’	Sigma-Aldrich®	Custom
*manet* R: 5’-GGTGAGGCTGCTTCATTTTC-3’	Sigma-Aldrich®	Custom
*nox5 ex4*.*4bp* F: 5’-GCTCAAGGGCTTACATGATCC-3’	Sigma-Aldrich®	Custom
*nox5 ex4*.*4bp* R: 5’-GCCTCAACATCAGCACCTAC-3’	Sigma-Aldrich®	Custom
M13 Reverse: 5’-GTAAAACGACGGCCAGTG-3’	Sigma-Aldrich®	Custom

### Resource availability

#### Lead contact

Further information and requests for resources and reagents should be directed to and will be fulfilled by the lead contact, Enrique Amaya (enrique.amaya@manchester.ac.uk).

#### Materials availability

Plasmids generated in this study are available upon request from the lead contact. Zebrafish lines generated in this study will be deposited to the European Zebrafish Resource Centre.

### Experimental model and subject details

The experimental model used for all *in vivo* experiments in this study was zebrafish (*Danio rerio*). All animal experiments were approved by the University of Manchester Animal Ethics Committee and were licenced in accordance with the Animals (Scientific Procedures) Act, 1986. Zebrafish husbandry was undertaken in a re-circulating system maintained at 28.5°C, with a 14hr photoperiod. These conditions are uniform for WT and all mutant strains. Embryos were obtained by marbling tanks, or by isolating pairs in breeding chambers. All experiments were carried out on random mixed sex cohorts. The age of the experimental animals used in this study ranged from 2 days post fertilisation (Figure S1) through to 22-month adults (Figures S3 and S4). Experiments focusing on comparing rate of caudal fin regeneration across different genotypes and/or HyPer imaging were carried out in adult animals between 6 and 8 months of age (Figures 3, 4 and 5, S2, S5-S8, S10 and S11). Data shown in Figure S9 was carried out in 3-month-old adults.

### Method details

#### Genomic extraction

Fin clips from caudal fins of individual adults were added to a mixture of lysis Buffer (10mM Tris-HCL, pH 8.0, 1mM EDTA, 0.3% Tween-20, 0.3% NP40), and proteinase K (20-25mg/ml) (New England Biolabs® Inc.), 1ul/50ul lysis buffer. This was incubated in a thermal cycler programmed to 55°C (2hours), 95°C (10 minutes) and a 12°C hold.

#### sgRNA design and production of CRISPR mutants

Single guide RNAs (sgRNAs) were designed for targeting exon 1 of *cyba*, and exon 4 of *nox5*, as previously described (Moreno-mateos et al. 2015). Briefly, the Ensemble ID for each gene when entered online on CRISPRscan generated multiple gRNAs. Exon-targeting gRNAs were then chosen based on rank, location within the first 50% of the ORF, and distance from the initiation codon. A sgRNA template requires a 52nt oligo (sgRNA primer) 5’ TAATACGACTCACTATAGG (N = 18) GTTTTAGAGCTAGAA, containing the T7 promoter, the 20nt specific DNA-binding sequence [GG(N = 18)] and a constant 15nt tail annealing sequence. This was annealed to an invariant 80nt reverse oligo AAAAGCACCGACTCGGTGCCACTTTTTCAAGTTGATAACGGACTAGCCTTATTTTAACTTGCTATTTCTAGCTCTAAAAC 3′ tail primer, generating a 117bp PCR product. Oligos were obtained from Sigma-Aldrich®. The PCR cycler settings for this primer extension were 3 min at 95°C; 30 cycles of 30s at 95°C, 30s at 45°C and 30s at 72°C; and a final step at 72°C for 7 min. PCR products were purified using Qiaquick (Qiagen) columns. Approximately 120–150ng of DNA was used as a template for a T7 *in vitro* transcription reaction. *In vitro* transcribed sgRNAs were treated with DNase and precipitated using sodium acetate and ethanol. Purified sgRNA and Cas9-NLS protein (New England Biolabs® Inc.) were diluted to 300ng/ul. Equal volumes of Cas9-NLS protein, sgRNA and Phenol Red (Sigma-Aldrich®) were mixed to obtain the final injection mix. Injection drop size was adjusted to 1nl using a graticule scale. All embryos were injected at the one-cell stage. F_1_ heterozygous animals were identified via restriction digest, and indels were characterised using Sanger sequencing.

#### ENU mutant strains and transgenic strains

ENU mutants for *cyba*^*sa11798*^ and *duox*^*sa9892*^ were discovered during the Zebrafish Mutation Project (Kettleborough et al., 2013). These fish were sourced from the European Zebrafish Resource Centre (EZRC). *The tshr^wpr23e1^* mutants (original name *manet^wpr23e1^*) were sourced from the laboratory of David Parichy (McMenamin et al. 2014). *cyba*^*sa11798*^ and *duox*^*sa9892*^ were identified via Sanger sequencing. *The tshr^wpr23e1^* mutants were identified via restriction digest. The *Tg(ubb*:*HyPer)* reporter line (*ubb* promoter driving HyPer) was generated by Tol2 transgenesis as previously described.^64^ Mutants and transgenics generated in the lab were given allele designation via ZFIN.

#### Polymerase chain reaction

PCRs were undertaken using ExTaq DNA Polymerase (TaKaRa). Primers (Sigma Aldrich) used are listed below.

**Table:** 

*cyba ex1*.*5bp* F	Genomic PCR	51.1°C
*cyba ex1*.*5bp* R		
*cyba sa11798* F	Genomic PCR	54°C
*cyba sa11798* R		
*duox sa9892* F	Genomic PCR	55°C
*duox sa9892* R		
*duox sa9892* seq	Sequencing	
*manet* F	Genomic PCR	54°C
*manet* R		
nox5 ex4.4bp F	Genomic PCR	55°C
nox5 ex4.4bp R		
M13 Reverse	Identifying indels	

#### *Aspergillus fumigatus* spore harvesting and microinjection

An *Aspergillus fumigatus* strain constitutively expressing the Turbo635 fluorescent protein in the A1163 background^65^ was used to infect the larvae. The strain was cultured on *Aspergillus* complete media (ACM) in a 20ml flask and incubated at 37°C for at least 2 days prior to spore harvesting. ACM (1L) was prepared using adenine (0.075g), glucose (10g), yeast extract (1g), bacteriological peptone (2g), casamino acids (1g), vitamin solution (10ml), salt solution (20ml), ammonium tartrate (10ml from 500mM stock), and 1.5% of agar. The pH was adjusted to 6.5 using 10M NaOH. Spores were harvested using a 0.5% NaCl / 0.002% Tween-20 solution (1X). 10ml of the 1X solution was added per flask and flasks were agitated to detach spores. This suspension was filtered through miracloth (Merck Millipore), followed by centrifugation at 4000RPM, for 5minutes. The resultant pellet was resuspended, followed again by centrifugation. Pellet was resuspended in 5ml of the 1X NaCl/Tween. 1/10 and 1/100 dilutions were made from this master solution using and were counted using a New Improved Neubauer haematocytometer (Marinfeld,Germany) on a Nikon Optiphot at a 40x magnification

Freshly harvested spores were used for each experiment. Prior to injection, larvae were anaesthetised in MS-222, and positioned laterally on injection plates made of 3% agarose in E3.^66^^,^^67^^,^^68^ A spore suspension containing 5 conidia/nl was directly injected into the yolk sac of 2dpf larvae, using a microinjection setup consisting of a Picospritzer II (General Valve Corporation) and a Leica MZ6 stereomicroscope. The injected volume was 1nl, unless specified otherwise. Viability of injected larvae was checked twice per day to monitor survival. Dead larvae were plated on Potato Dextrose Agar (PDA). Plates were incubated at 37°C and appearance of hyphal masses was used as confirmation for aspergillosis-led mortality.

#### Caudal fin amputation and regeneration

Fish were anaesthetised using 0.4 mg/mL (0.04%) MS-222 (Sigma Aldrich). Animals were imaged prior to amputation, at T0, 1, 2, 3, and 4weeks amputation (wpa), on a Leica M165 FC (Leica Microsystems). Using Photoshop CS5 (Adobe®), images were cropped at the level of the caudal peduncle, and fins were outlined using the brush tool. The “Record Measurements” command was used to obtain the total area of the outlined fin. Regeneration was calculated as a weekly increase, relative to the unamputated state, where the unamputated state was regarded as 100%. This is formulated as:

$$\frac{weekly\;fin\;size}{unamputated\;fin\;size}\;X\;100$$

Animals that had less than 40% or more than 55% of the caudal fin amputated were excluded from further analysis, as we wished to eliminate any potential differences in the rates of regeneration to be caused by differences how much fin had been amputated as has been shown previously by others.^39^^,^^40^^,^^41^

#### H_2_O_2_ detection

For visualising H_2_O_2_ levels in the caudal fin, fish were imaged on a high-end widefield microscope (Decon Vision) using a 4X objective. MetaMorph® software was used to setup the mircroscope to capture two wavelengths - HyPer low (CFP) emission 482/25 and HyPer high (YFP) emission 544/24. Exposure was set 1000 microseconds and binning was 1X1. Excitation was achieved using BP 430/24 and BP 500/20 filters. Emission was detected with a BP 535/30 filter. Animals were imaged before and after amputation, over a series of time points. To do this, they were anaesthetised as routine, placed upon 24mm X 50mm glass coverslips in a thin film of anaesthetic and mounted. Using ImageJ images were analysed by subtracting background, smoothing, formatting to 32-bit, then dividing to calculate the HyPer ratio. The average H_2_O_2_ over the area captured in the image was then calculated and visualised using Prism 8.1 (GraphPad Software, Inc.).

### Quantification and statistical analysis

MATLAB (MathWorks), SPSS (IBM Corporation) and GraphPad Prism 8.1 (GraphPad Software, Inc.) were used for all the statistical and computational tasks. All of the statistical details of experiments can be found in the figure legends. Null hypotheses were rejected when p value < 0.05. In all figure legends, n denotes the number of animals used for the experiment. The bar charts show the mean of the data with error bars representing the SD. The data was tested for normality, and normally distributed data was analysed using parametric tests (ANOVA and t-test). The measurements taken between different groups (WT and mutants) were independent. Equal variance of data was assumed as the residuals were found to be homoscedastic within groups. No outliers were found or excluded throughout the experiments.

Aspergillosis-related survival was statistically assessed using the Log Rank Test. For fin regeneration, comparisons were made using ANOVA (repeated measures and one-way) or t-test. Linear regressions were performed without interpolation, with a 95% significance level. The strength of the correlation was assessed by the correlation coefficient, adjusted R square. Where applicable, Pearson’s correlation coefficient and Spearman’s rank correlation coefficient was reported. For the unamputated fin, distribution was used for explaining the relationship between age and fin size. Oscillations were tested using power spectrum analysis based on Fourier transform as well as autocorrelation. MATLAB was used to perform power spectrum analysis and SSPS was used for autocorrelations.

## Data Availability

Any additional information required to reanalyze the data reported in this paper is available from the lead contact upon request.

## Supplemental information

Document S1. Figures S1–S11

Table S1. Variability in fin area measurements in triplicate

Table S2. Paired test showing difference in fin area measurements between fish
