# Supplementary material for: Duox is the primary NADPH oxidase responsible for ROS production during adult caudal fin regeneration in zebrafish
Source: iScience. 2023 Feb 4;26(3):106147. doi: 10.1016/j.isci.2023.106147 (PMC9950526; doi:10.1016/j.isci.2023.106147)
Supplement: Document S1. Figures S1–S11 [file mmc1.pdf]

iScience, Volume *26*

Supplemental information

Duox is the primary NADPH oxidase responsible for ROS production during adult caudal fin regeneration in zebrafish

Kunal Chopra, Milda Folkmanaitė, Liam Stockdale, Vishali Shathish, Shoko Ishibashi, Rachel Bergin, Jorge Amich, and Enrique Amaya

Figure S1

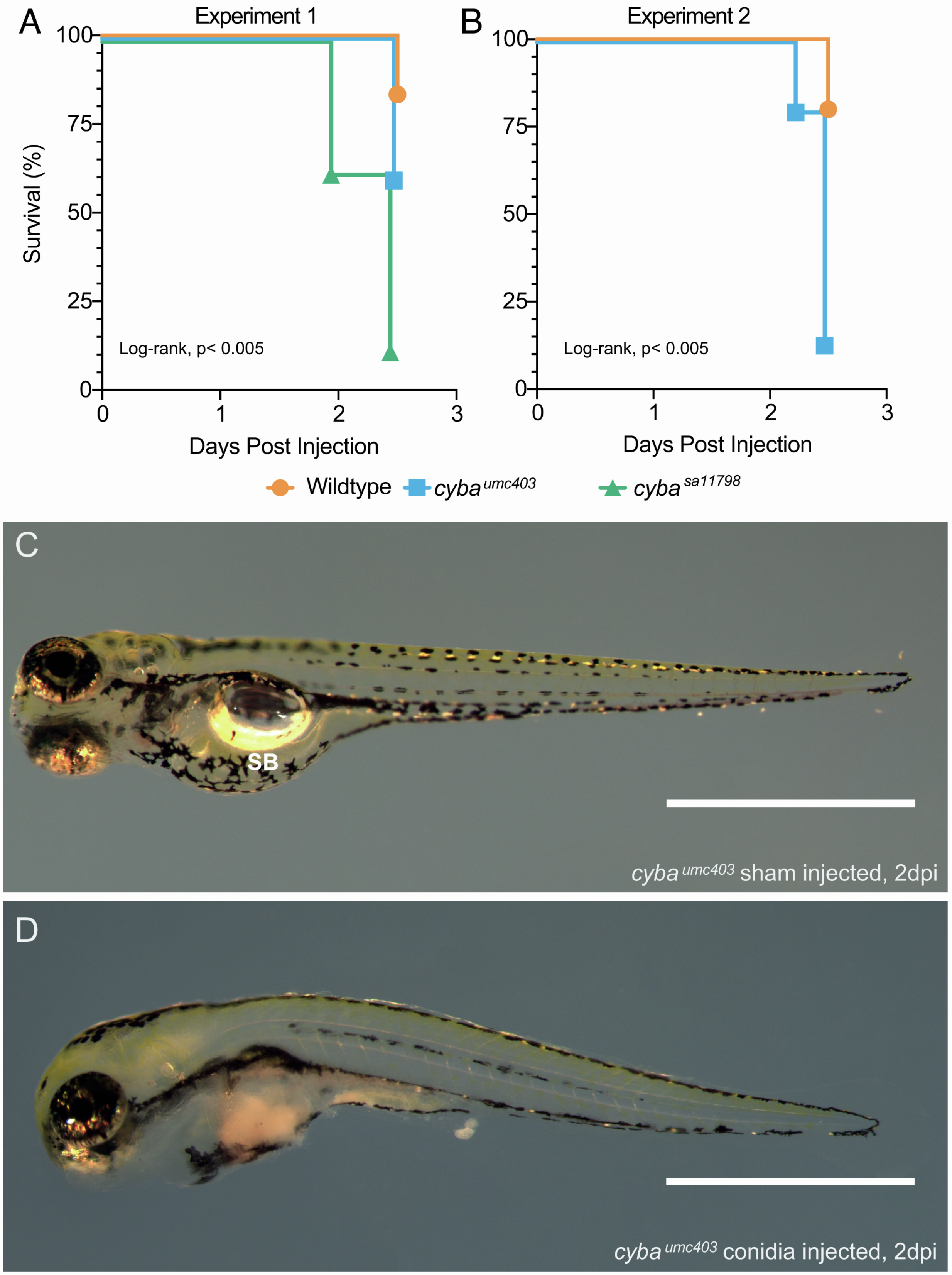

Figure S1. *cyba* mutants are highly vulnerable to aspergillosis. Related to Figure 1. (A and B) Survival of *cyba*^{umc403} and *cyba*^{sa11798} larvae was significantly affected following conidia injection (Log Rank Test, P< 0.005). (C and D) Mutant larvae show systemic infection following *Aspergillus* injection. Conidia injected animals show necroses and the swim bladder (SB) is not distinguishable at 2-3dpi. WT (n=17); *cyba*^{umc403} (n=25); *cyba*^{sa11798} (n=8). Scale bar in each image corresponds to 1 mm.

Figure S2

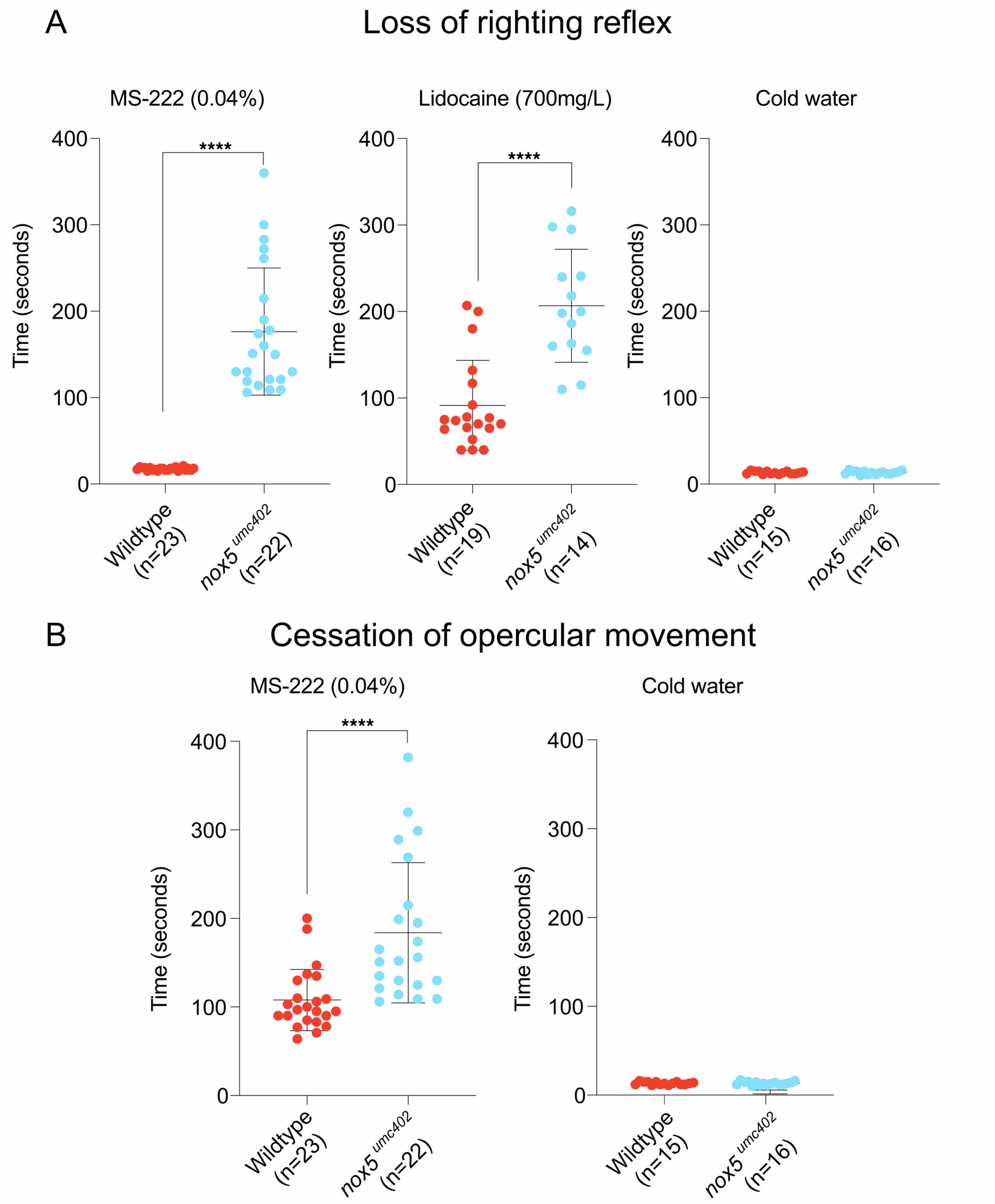

Figure S2. *nox5* mutants have a delayed response to some anaesthetics. Related to Figure 1. (A) *nox5*^{umc402} adult animals take significantly longer to respond to agents acting via ion channels, but not to cold water. For MS-222, WT (n=23); *nox5*^{umc402} (n=22), for Lidocaine, WT (n=19); *nox5*^{umc402} (n=14) and for cold water, WT (n=15); *nox5*^{umc402} (n=16). (B) Following immersion in MS-222 anaesthesia; WT (n=23); *nox5*^{umc402} (n=22), but not cold water; WT (n=15); *nox5*^{umc402} (n=16), opercular movement also continues for longer in *nox5*^{umc402} animals. Unpaired t test **** P< 0.0001.

Figure S3

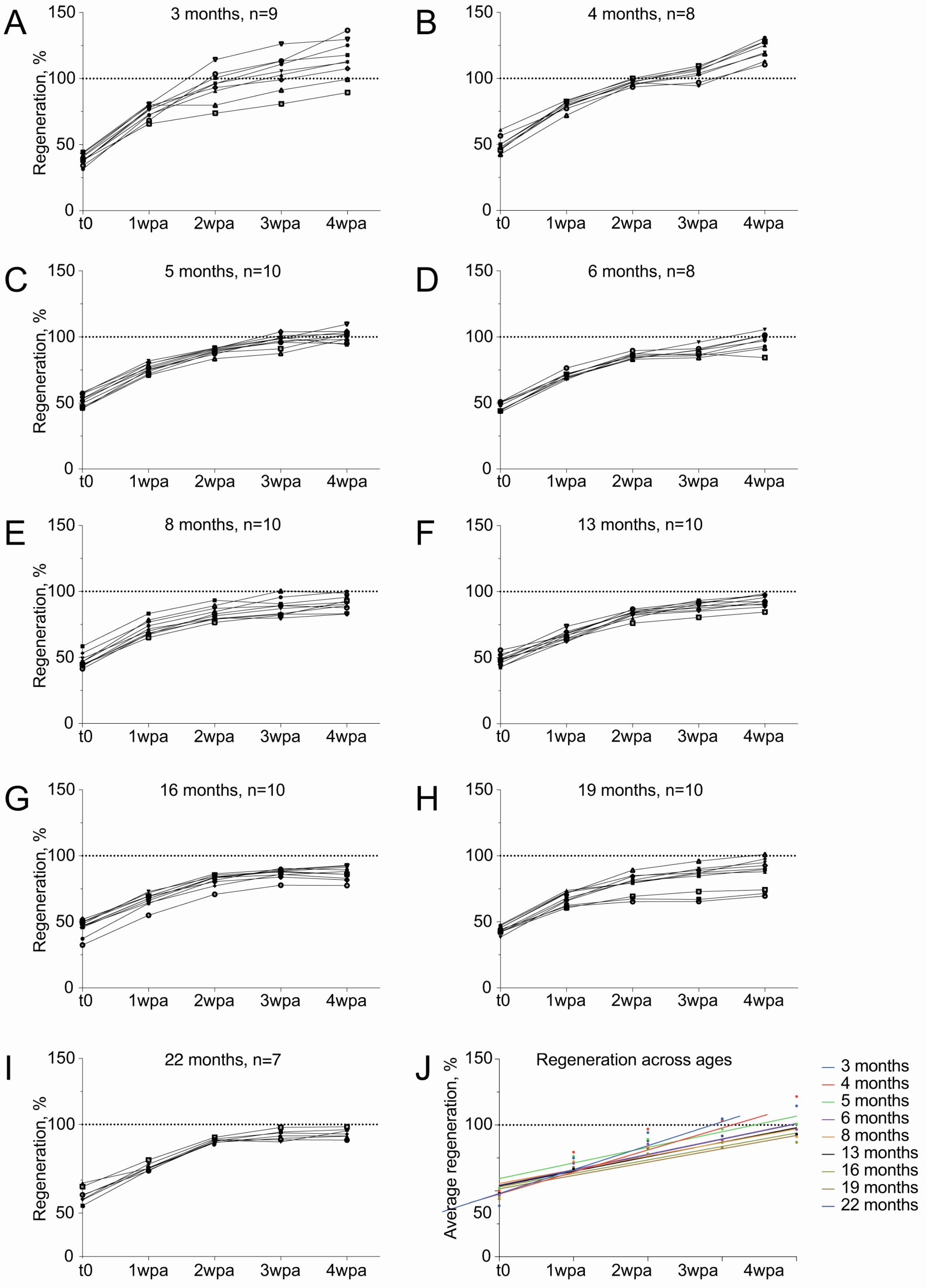

Figure S3. Qualitative representation of caudal fin regeneration amongst individual WT adults of various ages is represented using linear regression. Related to Figure 2. Younger animals, especially at 3 and 4 months of age (A and B) exceeded the original unamputated area by 4wpa, while 8 month old and older (E-I) animals regenerate to less than 100% of the original size by 4wpa. This is likely due to younger animals still growing in body size during their period of regeneration. Within each group, individual slopes were not significantly different. For 3 months (n=9), 4 months (n=8); 5 months (n=10), 6 months (n=8), 8 months (n=10), 13 months (n=10), 16 months (n=10), 19 months (n=10), 22 months (n=7).

Figure S4

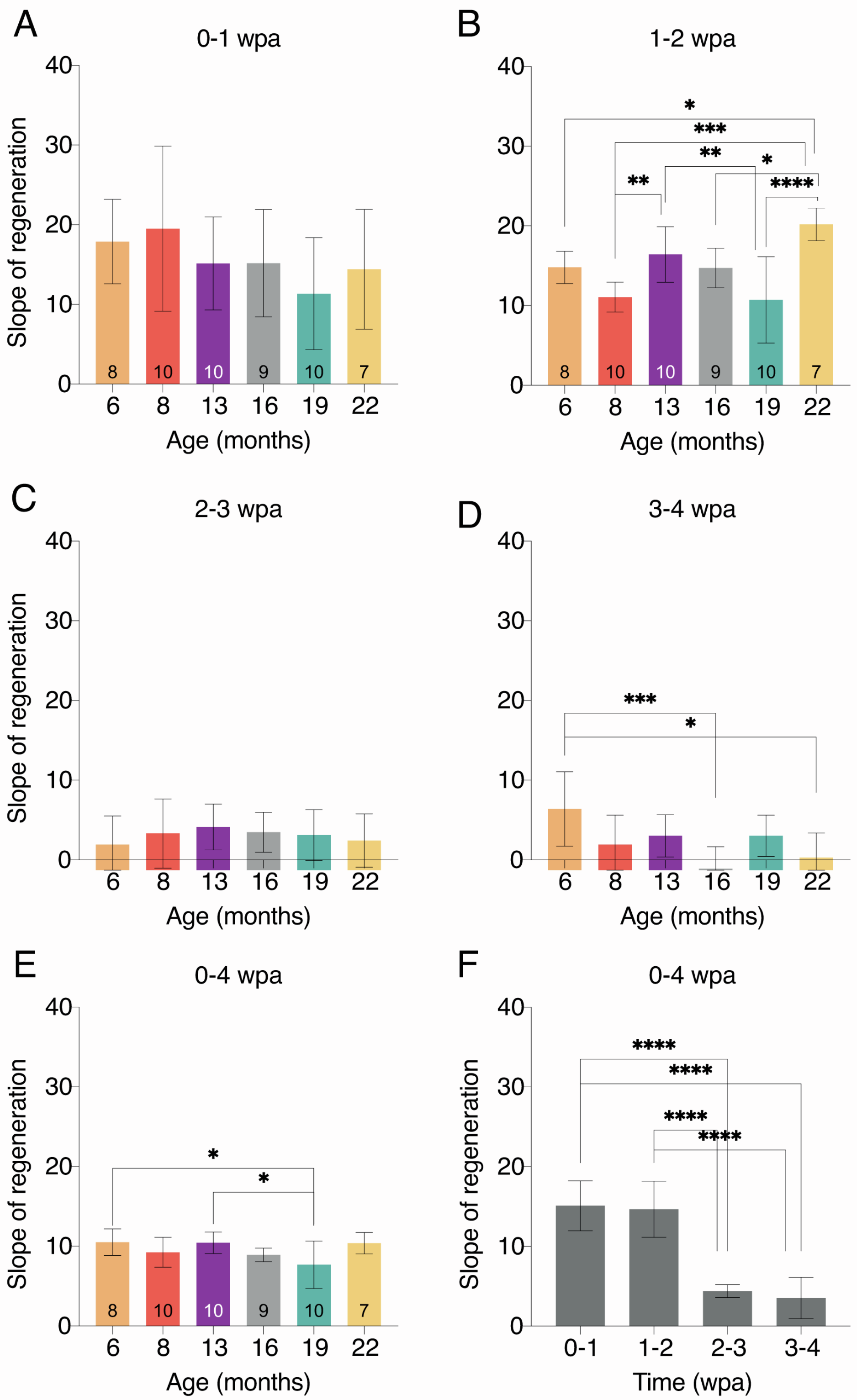

Figure S4. The effect of age on the rate of caudal fin regeneration. Related to Figure 2. No significant different in the rate of regeneration was seen in the first week (0-1) (A) and third week (2-3) (C), but significant differences in various age groups were observed during the second week (1-2) (B) and fourth week (3-4) (D) of regeneration. (E) Taken as a whole, differences in the rates of regeneration were non-significant between most age groups. Majority of the recovery is indicated to occur within the 2wpa, with significant differences recorded between the first 2 weeks and the latter 2 weeks. For 3 months (n=9), 4 months (n=8); 5 months (n=10), 6 months (n=8), 8 months (n=10), 13 months (n=10), 16 months (n=10), 19 months (n=10), 22 months (n=7), (Bonferroni's multiple comparisons test, *P<0.5, **P<0.01, ***P<0.001, ****P<0.0001)

Figure S5

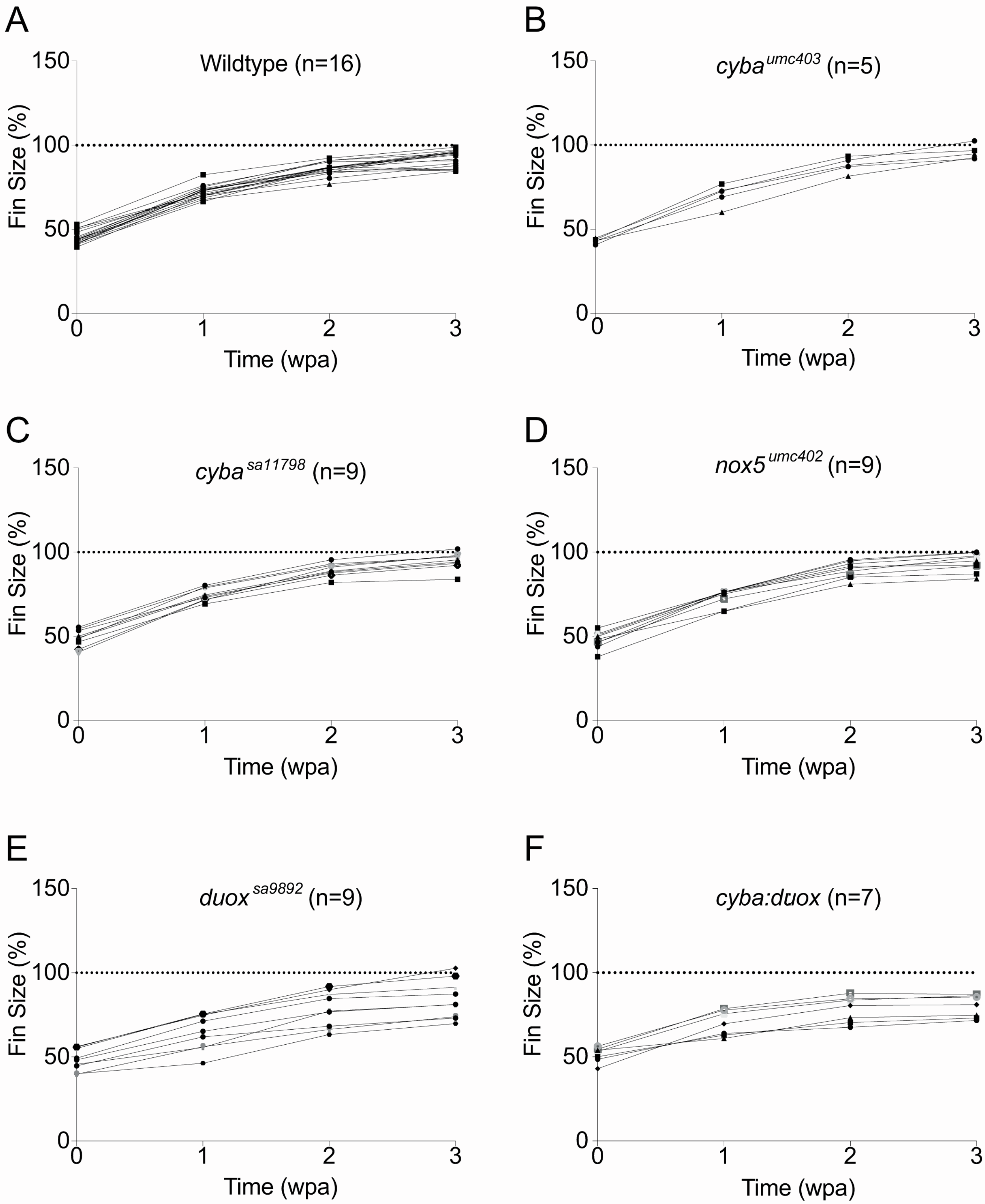

Figure S5. Quantitative representation of caudal fin regeneration amongst individual WT and *nox* mutant adults. Related to Figure 3. Over a three-week period, the majority of *duox*^{sa9892} (E) and *cyba:duox* fish (F) failed to complete regeneration of their fins. WT (n=16); *cyba*^{umc403} (n=5); *cyba*^{sa11798} (n=9); *nox5*^{umc402} (n=9); *duox*^{sa9892} (n=9); *cyba:duox* (n=7).

Figure S6

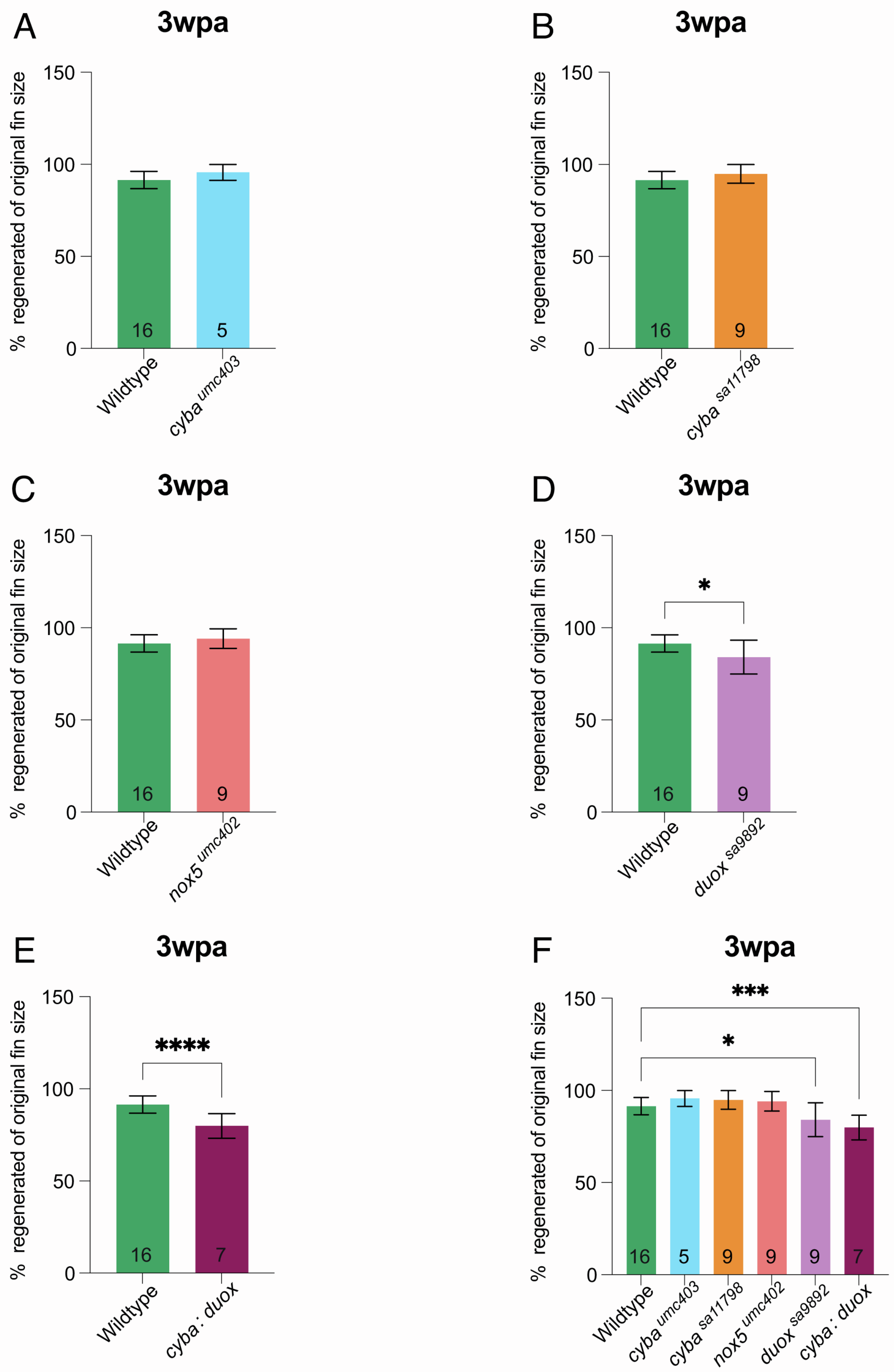

Figure S6. Overall caudal fin size at 3wpa in WT and *nox* mutants. Related to Figure 3. Unpaired t-test to compare the overall caudal fin size amongst WT and nox mutants (A-E) revealed significantly smaller fins amongst the *duox*^{sa9892} and *cyba:duox* animals (p<0.0001). This was further confirmed with one-way ANOVA comparing each nox mutant strain to WT (F) (p<0.0001). WT (n=16); *cyba*^{umc403} (n=5); *cyba*^{sa11798} (n=9); *nox5*^{umc402} (n=9); *duox*^{sa9892} (n=9); cyba:duox (n=7).

Figure S7

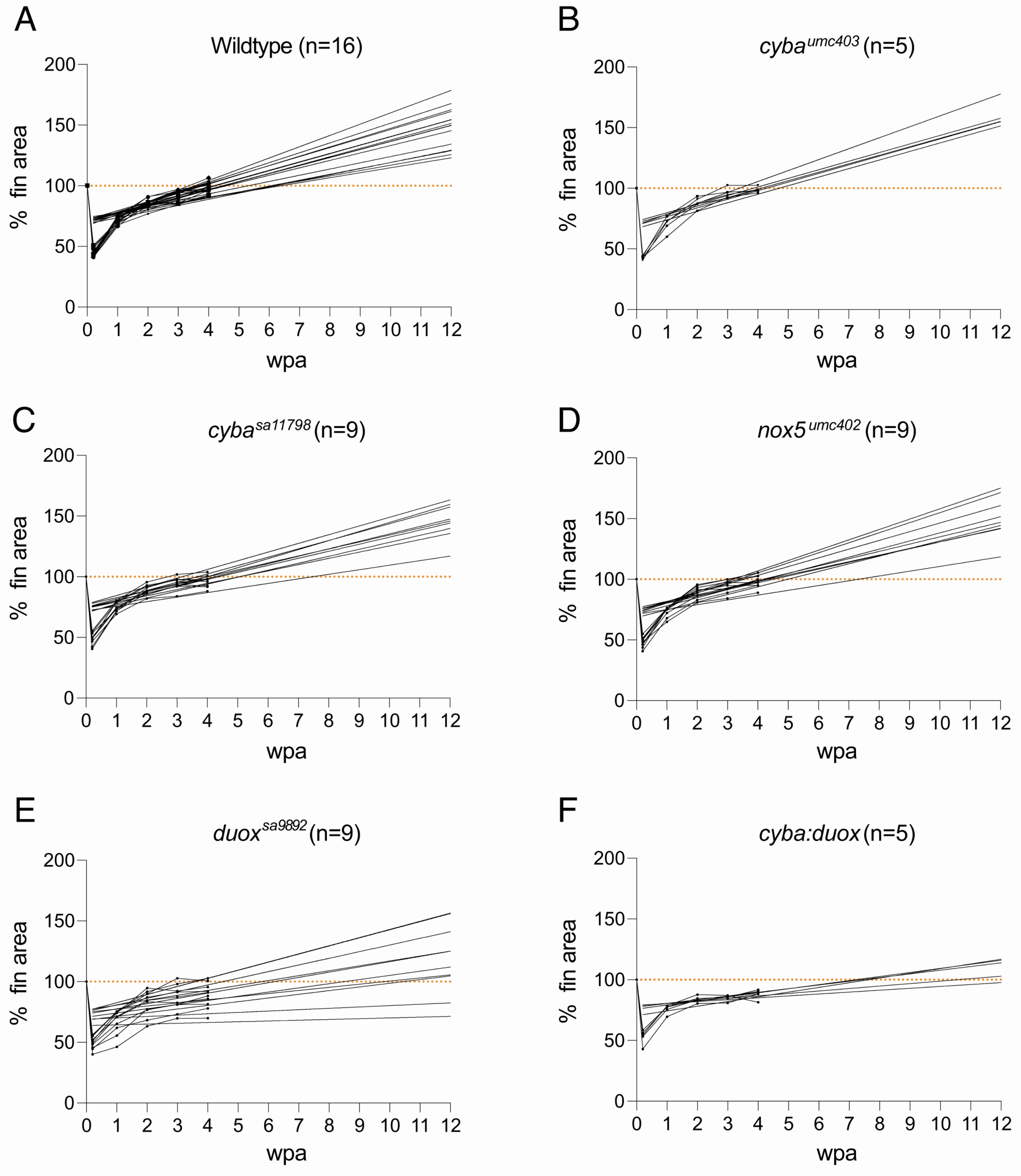

Figure S7. Projections for completion of caudal fin regeneration (wpa) in individual WT and *nox* mutant animals. Related to Figure 3. Nonlinear fit graphs illustrate that most WT individuals complete regeneration by 4wpa (A). Similar trends are observed amongst individual *cyba* and *nox5* mutants animals (B-D). In contrast, individual $duox^{sa9892}$ (E) and *cyba:duox* animals are predicted to take 5 to 12 weeks (in most cases) to complete regeneration. WT (n=16); $cyba^{umc403}$ (n=5); $cyba^{sa11798}$ (n=9); $nox5^{umc402}$ (n=9); $duox^{sa9892}$ (n=9); cyba:duox (n=5).

Figure S8

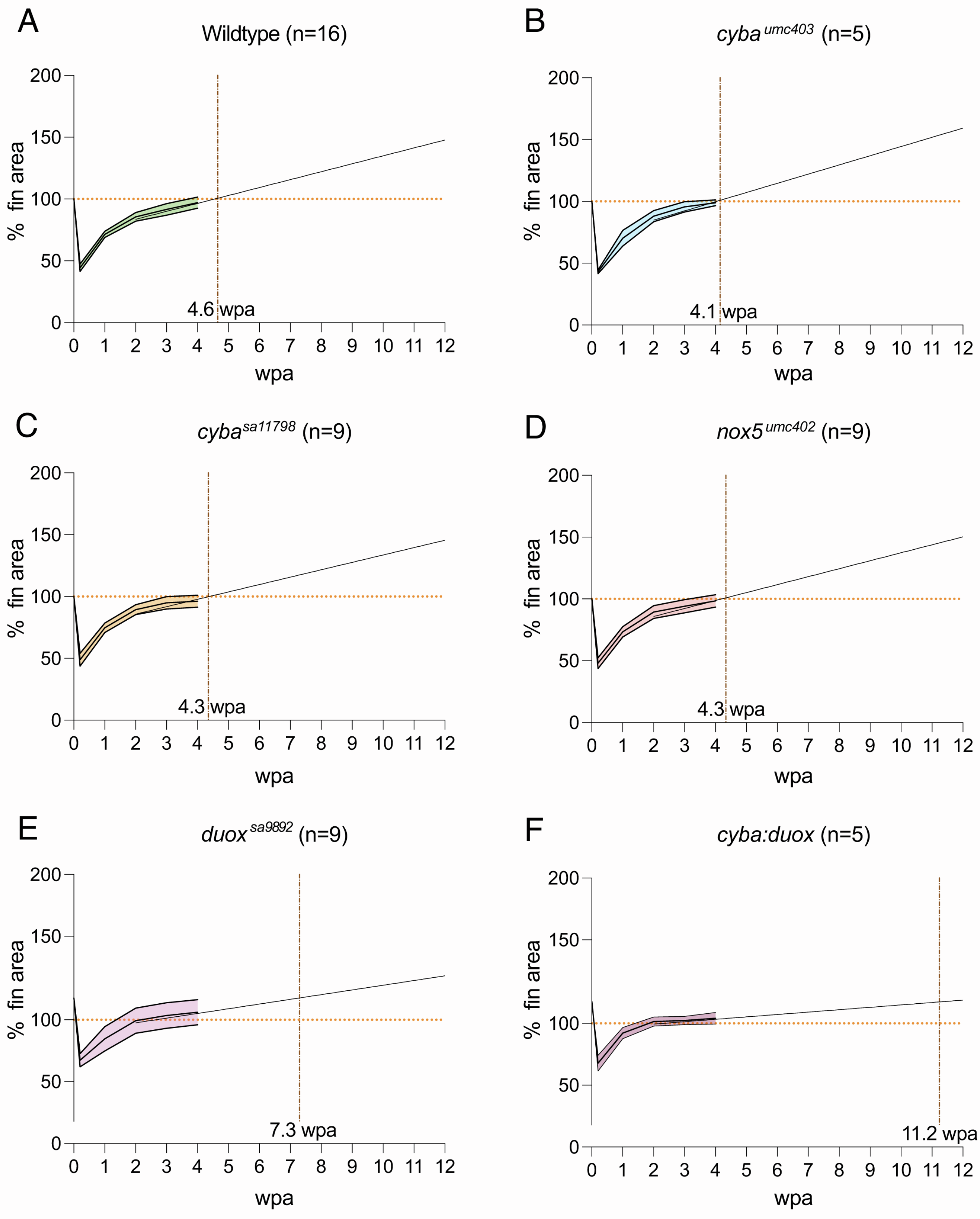

Figure S8. Projections for the averages of completion of caudal fin regeneration (wpa) in WT and *nox* mutant animals. Related to Figure 3. Row statistics with slopes derived from simple linear regression allow extrapolation to the time when the regenerating fin would reach the original unamputated size. WTs, *cyba* and *nox5* mutants complete regeneration before 4wpa (A-D). In contrast, *duox*^{sa9892} (E) and *cyba:duox* (F) animals are predicted to take 7 to 11 weeks to complete regeneration. Shaded areas represent the extent of the error bars for mean. Dashed vertical lines represent the time point when regeneration reaches 100%. WT (n=16); *cyba*^{umc403} (n=5); *cyba*^{sa11798} (n=9); *nox5*^{umc402} (n=9); *duox*^{sa9892} (n=9); *cyba:duox* (n=5).

Figure S9

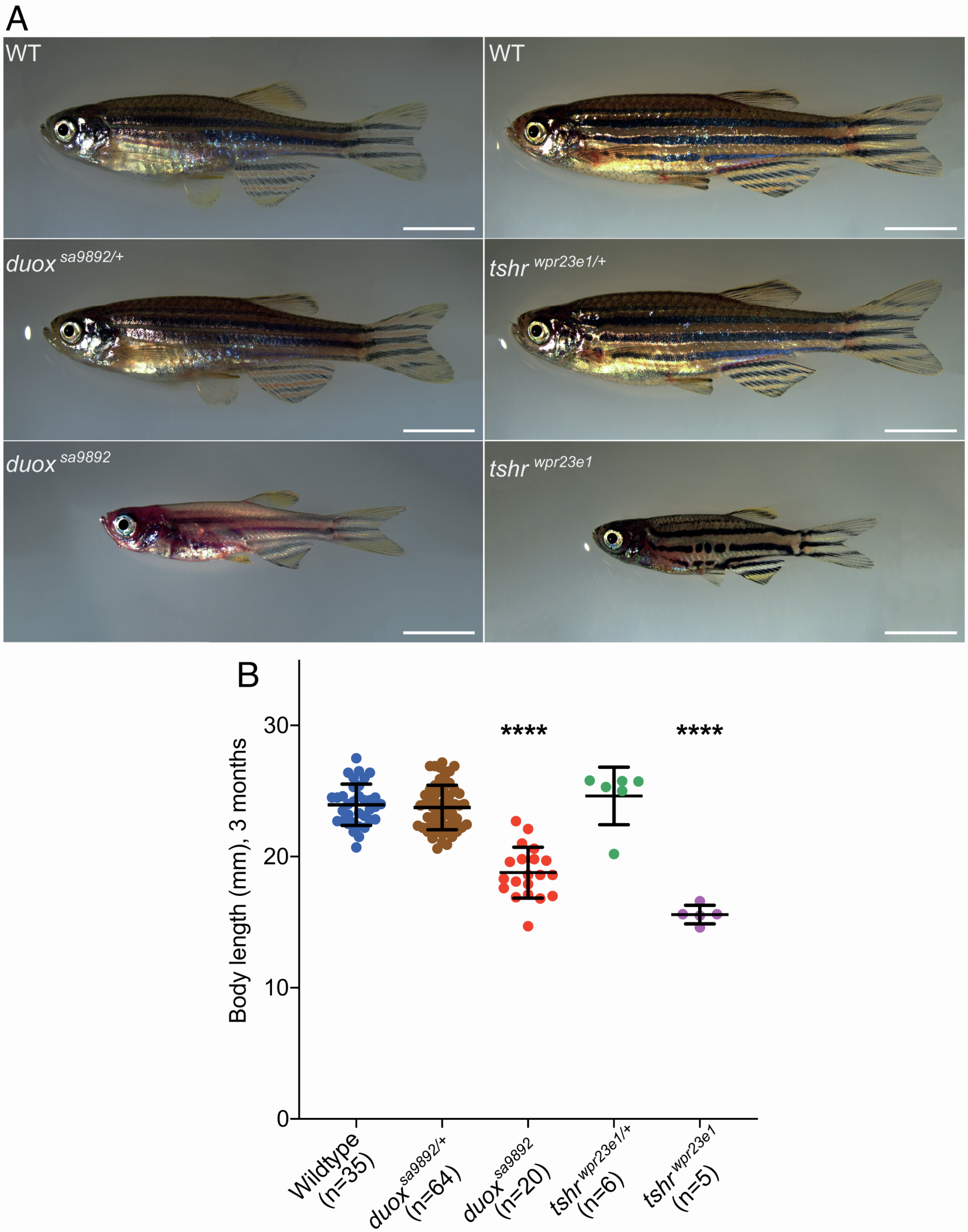

Figure S9. External phenotypes are concordant amongst *duox* and *tsrh* mutant animals. Related to Figure 4. (A) duox^{sa9892}/+ and tshr^{wpr23e1}/+ zebrafish are similar to WT siblings in terms of body-length, at 3 months of age, while duox^{sa9892} and tshr^{wpr23e1} animals are significantly shorter than all other groups. Scale bar in each image corresponds to 1cm. (B) Quantification of body lengths of WT (n=35), duox^{sa9892}/+ (n=64), duox^{sa9892} (n=20), tshr^{wpr23e1}/+ (n=6) and tshr^{wpr23e1} homozygous (n=5) fish at 3 months reveals a significantly shorter body length in the duox^{sa9892} and tshr^{wpr23e1}, relative to the other genotypes (Bonferroni's multiple comparisons test, ****P<0.0001).

Figure S10

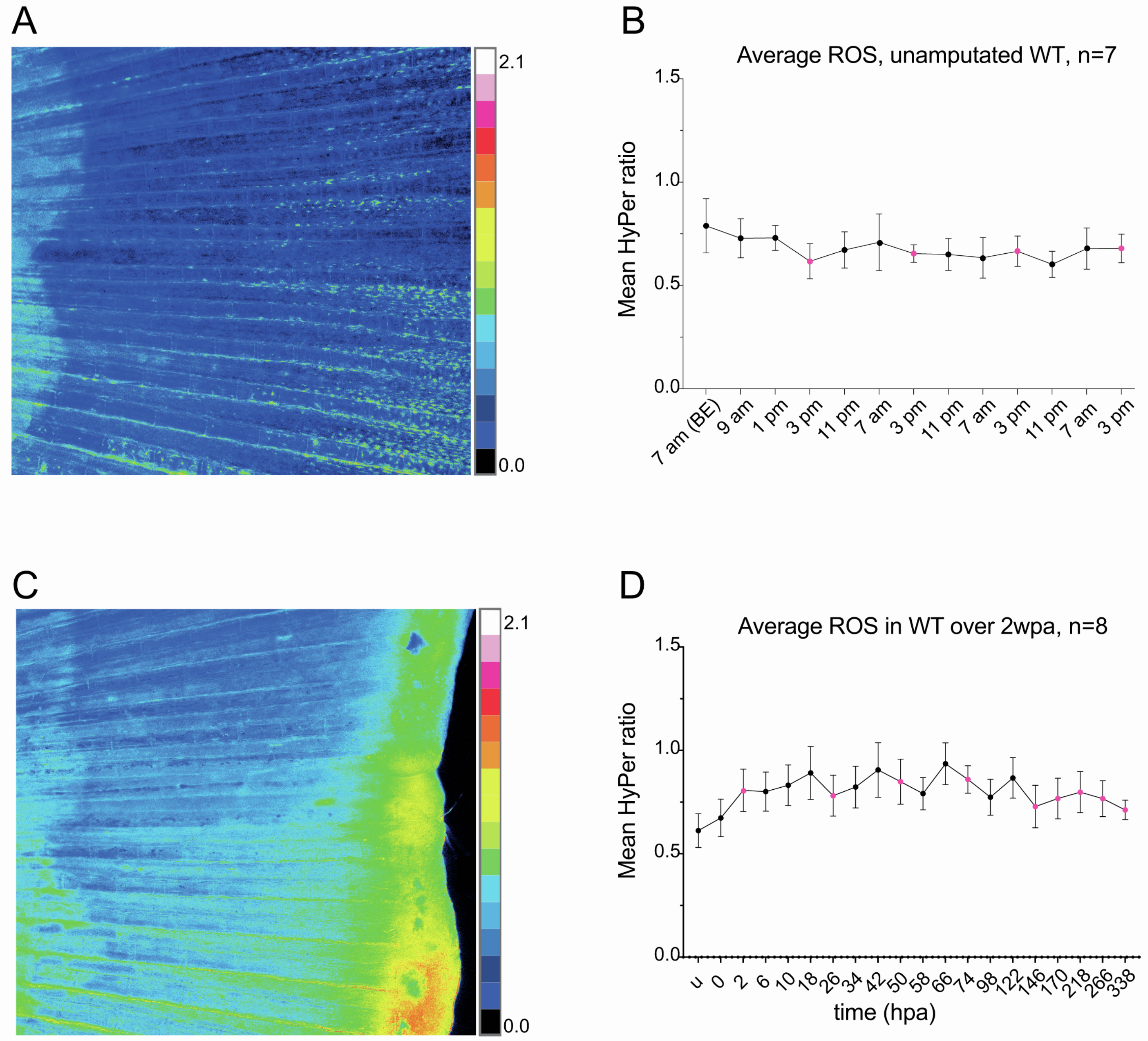

Figure S10. HyPer ratios in unamputated and amputated Tg(ubb:HyPer)umc400 adult fins over time. Related to Figure 5. (A-B) Hyper ratio is low and remains low over time in unamputated WT fins (n=7). (C-D) Hyper ratios rise following amputation and remain higher than baseline levels for up to two weeks following injury (n=8).

Figure S11

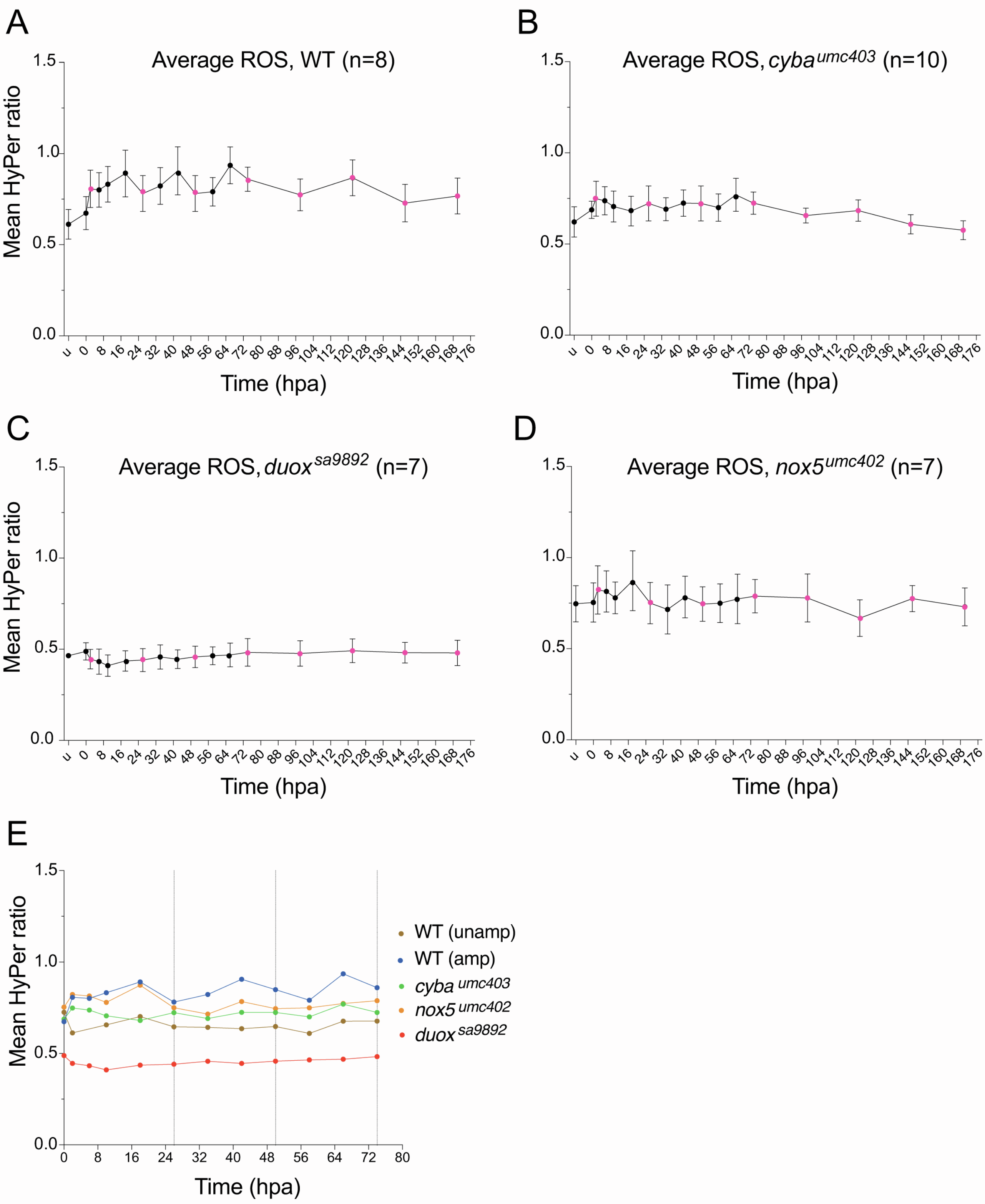

Figure S11. Average HyPer ratios following amputation in WT and *nox* mutant adult fins over a week (168hpa). nox mutants were generated in a HyPer transgenic background. Related to Figure 5. Average Hyper ratios in (A) WT (n=8), (B) *cyba*^{umc403} (n=10), (C) *duox*^{sa9892} (n=7) and (D) *nox5*^{umc402} (n=7) adult fins. Pink dots correlate with ROS levels at 3pm each day. (E) Average Hyper ratios of all different genotypes shown in the same graph, revealing that *duox*^{sa9892} animals have a significantly lower ROS levels than the other genotypes and also lower than unamputated WT fins.
