# Supplementary material for: Duox is the primary NADPH oxidase responsible for ROS production during adult caudal fin regeneration in zebrafish
Source: iScience. 2023 Feb 4;26(3):106147. doi: 10.1016/j.isci.2023.106147 (PMC9950526; doi:10.1016/j.isci.2023.106147)
Supplement: Table S1. Variability in fin area measurements in triplicate [file mmc2.docx]

Table S1. Variability in fin area measurements in triplicate

|  | **fish1** | **fish2** | **fish3** | **fish4** | **fish5** | **fish6** | **fish7** | **fish8** | **fish9** | **fish10** | **fish11** | **fish12** | **fish13** | **fish14** | **fish15** | **fish16** | **fish17** | **fish18** |
| --- | --- | --- | --- | --- | --- | --- | --- | --- | --- | --- | --- | --- | --- | --- | --- | --- | --- | --- |
| **Number of values** | 3 | 3 | 3 | 3 | 3 | 3 | 3 | 3 | 3 | 3 | 3 | 3 | 3 | 3 | 3 | 3 | 3 | 3 |
|  |  |  |  |  |  |  |  |  |  |  |  |  |  |  |  |  |  |  |
| **Minimum** | 381839 | 350571 | 304221 | 351764 | 368015 | 372662 | 416264 | 355717 | 299816 | 320440 | 391288 | 358304 | 385499 | 396845 | 326099 | 404817 | 341676 | 307412 |
| **Maximum** | 390738 | 367148 | 315706 | 364775 | 385293 | 402233 | 432525 | 376494 | 310024 | 334221 | 401428 | 371192 | 395682 | 398752 | 338040 | 418770 | 354591 | 311579 |
| **Range** | 8899 | 16577 | 11485 | 13011 | 17278 | 29571 | 16261 | 20777 | 10208 | 13781 | 10140 | 12888 | 10183 | 1907 | 11941 | 13953 | 12915 | 4167 |
|  |  |  |  |  |  |  |  |  |  |  |  |  |  |  |  |  |  |  |
| **Mean** | 384885 | 360344 | 311318 | 357922 | 379291 | 391887 | 426710 | 362965 | 305227 | 325776 | 395141 | 362829 | 390108 | 398028 | 333711 | 409743 | 349569 | 309623 |
| **Std. Deviation** | 5070 | 8678 | 6203 | 6533 | 9772 | 16665 | 9066 | 11726 | 5132 | 7398 | 5491 | 7250 | 5160 | 1033 | 6613 | 7828 | 6919 | 2095 |
| **Std. Error of Mean** | 2927 | 5010 | 3581 | 3772 | 5642 | 9622 | 5234 | 6770 | 2963 | 4271 | 3170 | 4186 | 2979 | 596.3 | 3818 | 4520 | 3995 | 1210 |
|  |  |  |  |  |  |  |  |  |  |  |  |  |  |  |  |  |  |  |
| **Lower 95% CI of mean** | 372291 | 338786 | 295908 | 341692 | 355015 | 350488 | 404189 | 333835 | 292479 | 307399 | 381500 | 344818 | 377290 | 395462 | 317284 | 390296 | 332380 | 304418 |
| **Upper 95% CI of mean** | 397480 | 381903 | 326728 | 374152 | 403567 | 433285 | 449232 | 392095 | 317974 | 344153 | 408781 | 380840 | 402925 | 400593 | 350138 | 429190 | 366757 | 314827 |
|  |  |  |  |  |  |  |  |  |  |  |  |  |  |  |  |  |  |  |
| **Are all 3 measurements within the CI?** | TRUE | TRUE | TRUE | TRUE | TRUE | TRUE | TRUE | TRUE | TRUE | TRUE | TRUE | TRUE | TRUE | TRUE | TRUE | TRUE | TRUE | TRUE |
