# Supplementary material for: Duox is the primary NADPH oxidase responsible for ROS production during adult caudal fin regeneration in zebrafish
Source: iScience. 2023 Feb 4;26(3):106147. doi: 10.1016/j.isci.2023.106147 (PMC9950526; doi:10.1016/j.isci.2023.106147)
Supplement: Table S2. Paired test showing difference in fin area measurements between fish [file mmc3.docx]

Table S2. Paired test showing difference in fin area measurements between fish

|  |  |  |  |  |  |
| --- | --- | --- | --- | --- | --- |
| **Number of families** | 1 |  |  |  |  |
| **Number of comparisons per family** | 153 |  |  |  |  |
| **Alpha** | 0.05 |  |  |  |  |
|  |  |  |  |  |  |
| **Bonferroni's multiple comparisons test** | Mean Diff. | 95.00% CI of diff. | Significant? | Summary | Adjusted P Value |
| **fish1 vs. fish2** | 24541 | -1088 to 50170 | No | ns | 0.0814 |
| **fish1 vs. fish3** | 73567 | 47939 to 99196 | Yes | **** | <0.0001 |
| **fish1 vs. fish4** | 26963 | 1335 to 52592 | Yes | * | 0.0272 |
| **fish1 vs. fish5** | 5594 | -20035 to 31223 | No | ns | >0.9999 |
| **fish1 vs. fish6** | -7001 | -32630 to 18627 | No | ns | >0.9999 |
| **fish1 vs. fish7** | -41825 | -67454 to -16196 | Yes | **** | <0.0001 |
| **fish1 vs. fish8** | 21920 | -3708 to 47549 | No | ns | 0.2559 |
| **fish1 vs. fish9** | 79659 | 54030 to 105287 | Yes | **** | <0.0001 |
| **fish1 vs. fish10** | 59109 | 33481 to 84738 | Yes | **** | <0.0001 |
| **fish1 vs. fish11** | -10255 | -35884 to 15373 | No | ns | >0.9999 |
| **fish1 vs. fish12** | 22056 | -3573 to 47685 | No | ns | 0.2415 |
| **fish1 vs. fish13** | -5222 | -30851 to 20406 | No | ns | >0.9999 |
| **fish1 vs. fish14** | -13142 | -38771 to 12486 | No | ns | >0.9999 |
| **fish1 vs. fish15** | 51174 | 25546 to 76803 | Yes | **** | <0.0001 |
| **fish1 vs. fish16** | -24858 | -50486 to 770.8 | No | ns | 0.0707 |
| **fish1 vs. fish17** | 35317 | 9688 to 60945 | Yes | *** | 0.0005 |
| **fish1 vs. fish18** | 75263 | 49634 to 100891 | Yes | **** | <0.0001 |
| **fish2 vs. fish3** | 49026 | 23398 to 74655 | Yes | **** | <0.0001 |
| **fish2 vs. fish4** | 2422 | -23206 to 28051 | No | ns | >0.9999 |
| **fish2 vs. fish5** | -18947 | -44576 to 6682 | No | ns | 0.8799 |
| **fish2 vs. fish6** | -31542 | -57171 to -5914 | Yes | ** | 0.0032 |
| **fish2 vs. fish7** | -66366 | -91995 to -40737 | Yes | **** | <0.0001 |
| **fish2 vs. fish8** | -2621 | -28249 to 23008 | No | ns | >0.9999 |
| **fish2 vs. fish9** | 55118 | 29489 to 80746 | Yes | **** | <0.0001 |
| **fish2 vs. fish10** | 34568 | 8940 to 60197 | Yes | *** | 0.0008 |
| **fish2 vs. fish11** | -34796 | -60425 to -9168 | Yes | *** | 0.0007 |
| **fish2 vs. fish12** | -2485 | -28114 to 23144 | No | ns | >0.9999 |
| **fish2 vs. fish13** | -29763 | -55392 to -4135 | Yes | ** | 0.0074 |
| **fish2 vs. fish14** | -37683 | -63312 to -12055 | Yes | *** | 0.0002 |
| **fish2 vs. fish15** | 26633 | 1005 to 52262 | Yes | * | 0.0317 |
| **fish2 vs. fish16** | -49399 | -75027 to -23770 | Yes | **** | <0.0001 |
| **fish2 vs. fish17** | 10776 | -14853 to 36404 | No | ns | >0.9999 |
| **fish2 vs. fish18** | 50722 | 25093 to 76350 | Yes | **** | <0.0001 |
| **fish3 vs. fish4** | -46604 | -72233 to -20975 | Yes | **** | <0.0001 |
| **fish3 vs. fish5** | -67973 | -93602 to -42345 | Yes | **** | <0.0001 |
| **fish3 vs. fish6** | -80569 | -106197 to -54940 | Yes | **** | <0.0001 |
| **fish3 vs. fish7** | -115392 | -141021 to -89764 | Yes | **** | <0.0001 |
| **fish3 vs. fish8** | -51647 | -77276 to -26018 | Yes | **** | <0.0001 |
| **fish3 vs. fish9** | 6091 | -19537 to 31720 | No | ns | >0.9999 |
| **fish3 vs. fish10** | -14458 | -40087 to 11171 | No | ns | >0.9999 |
| **fish3 vs. fish11** | -83823 | -109451 to -58194 | Yes | **** | <0.0001 |
| **fish3 vs. fish12** | -51511 | -77140 to -25883 | Yes | **** | <0.0001 |
| **fish3 vs. fish13** | -78790 | -104418 to -53161 | Yes | **** | <0.0001 |
| **fish3 vs. fish14** | -86710 | -112338 to -61081 | Yes | **** | <0.0001 |
| **fish3 vs. fish15** | -22393 | -48022 to 3236 | No | ns | 0.2089 |
| **fish3 vs. fish16** | -98425 | -124054 to -72796 | Yes | **** | <0.0001 |
| **fish3 vs. fish17** | -38251 | -63879 to -12622 | Yes | *** | 0.0001 |
| **fish3 vs. fish18** | 1695 | -23933 to 27324 | No | ns | >0.9999 |
| **fish4 vs. fish5** | -21369 | -46998 to 4259 | No | ns | 0.3236 |
| **fish4 vs. fish6** | -33965 | -59593 to -8336 | Yes | ** | 0.0010 |
| **fish4 vs. fish7** | -68788 | -94417 to -43160 | Yes | **** | <0.0001 |
| **fish4 vs. fish8** | -5043 | -30672 to 20586 | No | ns | >0.9999 |
| **fish4 vs. fish9** | 52695 | 27067 to 78324 | Yes | **** | <0.0001 |
| **fish4 vs. fish10** | 32146 | 6517 to 57775 | Yes | ** | 0.0024 |
| **fish4 vs. fish11** | -37219 | -62847 to -11590 | Yes | *** | 0.0002 |
| **fish4 vs. fish12** | -4907 | -30536 to 20721 | No | ns | >0.9999 |
| **fish4 vs. fish13** | -32186 | -57814 to -6557 | Yes | ** | 0.0024 |
| **fish4 vs. fish14** | -40106 | -65734 to -14477 | Yes | **** | <0.0001 |
| **fish4 vs. fish15** | 24211 | -1418 to 49840 | No | ns | 0.0943 |
| **fish4 vs. fish16** | -51821 | -77450 to -26192 | Yes | **** | <0.0001 |
| **fish4 vs. fish17** | 8353 | -17275 to 33982 | No | ns | >0.9999 |
| **fish4 vs. fish18** | 48299 | 22671 to 73928 | Yes | **** | <0.0001 |
| **fish5 vs. fish6** | -12595 | -38224 to 13033 | No | ns | >0.9999 |
| **fish5 vs. fish7** | -47419 | -73048 to -21790 | Yes | **** | <0.0001 |
| **fish5 vs. fish8** | 16326 | -9302 to 41955 | No | ns | >0.9999 |
| **fish5 vs. fish9** | 74065 | 48436 to 99693 | Yes | **** | <0.0001 |
| **fish5 vs. fish10** | 53515 | 27887 to 79144 | Yes | **** | <0.0001 |
| **fish5 vs. fish11** | -15849 | -41478 to 9779 | No | ns | >0.9999 |
| **fish5 vs. fish12** | 16462 | -9167 to 42091 | No | ns | >0.9999 |
| **fish5 vs. fish13** | -10816 | -36445 to 14812 | No | ns | >0.9999 |
| **fish5 vs. fish14** | -18736 | -44365 to 6892 | No | ns | 0.9574 |
| **fish5 vs. fish15** | 45580 | 19952 to 71209 | Yes | **** | <0.0001 |
| **fish5 vs. fish16** | -30452 | -56080 to -4823 | Yes | ** | 0.0054 |
| **fish5 vs. fish17** | 29723 | 4094 to 55351 | Yes | ** | 0.0076 |
| **fish5 vs. fish18** | 69669 | 44040 to 95297 | Yes | **** | <0.0001 |
| **fish6 vs. fish7** | -34824 | -60452 to -9195 | Yes | *** | 0.0007 |
| **fish6 vs. fish8** | 28922 | 3293 to 54550 | Yes | * | 0.0110 |
| **fish6 vs. fish9** | 86660 | 61031 to 112289 | Yes | **** | <0.0001 |
| **fish6 vs. fish10** | 66111 | 40482 to 91739 | Yes | **** | <0.0001 |
| **fish6 vs. fish11** | -3254 | -28883 to 22375 | No | ns | >0.9999 |
| **fish6 vs. fish12** | 29057 | 3429 to 54686 | Yes | * | 0.0104 |
| **fish6 vs. fish13** | 1779 | -23850 to 27408 | No | ns | >0.9999 |
| **fish6 vs. fish14** | -6141 | -31770 to 19488 | No | ns | >0.9999 |
| **fish6 vs. fish15** | 58176 | 32547 to 83804 | Yes | **** | <0.0001 |
| **fish6 vs. fish16** | -17856 | -43485 to 7772 | No | ns | >0.9999 |
| **fish6 vs. fish17** | 42318 | 16689 to 67947 | Yes | **** | <0.0001 |
| **fish6 vs. fish18** | 82264 | 56635 to 107893 | Yes | **** | <0.0001 |
| **fish7 vs. fish8** | 63745 | 38117 to 89374 | Yes | **** | <0.0001 |
| **fish7 vs. fish9** | 121484 | 95855 to 147112 | Yes | **** | <0.0001 |
| **fish7 vs. fish10** | 100934 | 75306 to 126563 | Yes | **** | <0.0001 |
| **fish7 vs. fish11** | 31570 | 5941 to 57198 | Yes | ** | 0.0032 |
| **fish7 vs. fish12** | 63881 | 38252 to 89510 | Yes | **** | <0.0001 |
| **fish7 vs. fish13** | 36603 | 10974 to 62231 | Yes | *** | 0.0003 |
| **fish7 vs. fish14** | 28683 | 3054 to 54311 | Yes | * | 0.0123 |
| **fish7 vs. fish15** | 92999 | 67371 to 118628 | Yes | **** | <0.0001 |
| **fish7 vs. fish16** | 16967 | -8661 to 42596 | No | ns | >0.9999 |
| **fish7 vs. fish17** | 77142 | 51513 to 102770 | Yes | **** | <0.0001 |
| **fish7 vs. fish18** | 117088 | 91459 to 142716 | Yes | **** | <0.0001 |
| **fish8 vs. fish9** | 57738 | 32110 to 83367 | Yes | **** | <0.0001 |
| **fish8 vs. fish10** | 37189 | 11560 to 62818 | Yes | *** | 0.0002 |
| **fish8 vs. fish11** | -32176 | -57804 to -6547 | Yes | ** | 0.0024 |
| **fish8 vs. fish12** | 135.7 | -25493 to 25764 | No | ns | >0.9999 |
| **fish8 vs. fish13** | -27143 | -52771 to -1514 | Yes | * | 0.0251 |
| **fish8 vs. fish14** | -35063 | -60691 to -9434 | Yes | *** | 0.0006 |
| **fish8 vs. fish15** | 29254 | 3625 to 54883 | Yes | ** | 0.0094 |
| **fish8 vs. fish16** | -46778 | -72407 to -21149 | Yes | **** | <0.0001 |
| **fish8 vs. fish17** | 13396 | -12232 to 39025 | No | ns | >0.9999 |
| **fish8 vs. fish18** | 53342 | 27714 to 78971 | Yes | **** | <0.0001 |
| **fish9 vs. fish10** | -20549 | -46178 to 5079 | No | ns | 0.4566 |
| **fish9 vs. fish11** | -89914 | -115543 to -64285 | Yes | **** | <0.0001 |
| **fish9 vs. fish12** | -57603 | -83231 to -31974 | Yes | **** | <0.0001 |
| **fish9 vs. fish13** | -84881 | -110510 to -59252 | Yes | **** | <0.0001 |
| **fish9 vs. fish14** | -92801 | -118430 to -67172 | Yes | **** | <0.0001 |
| **fish9 vs. fish15** | -28484 | -54113 to -2856 | Yes | * | 0.0135 |
| **fish9 vs. fish16** | -104516 | -130145 to -78888 | Yes | **** | <0.0001 |
| **fish9 vs. fish17** | -44342 | -69971 to -18713 | Yes | **** | <0.0001 |
| **fish9 vs. fish18** | -4396 | -30025 to 21233 | No | ns | >0.9999 |
| **fish10 vs. fish11** | -69365 | -94993 to -43736 | Yes | **** | <0.0001 |
| **fish10 vs. fish12** | -37053 | -62682 to -11425 | Yes | *** | 0.0002 |
| **fish10 vs. fish13** | -64332 | -89960 to -38703 | Yes | **** | <0.0001 |
| **fish10 vs. fish14** | -72252 | -97880 to -46623 | Yes | **** | <0.0001 |
| **fish10 vs. fish15** | -7935 | -33564 to 17694 | No | ns | >0.9999 |
| **fish10 vs. fish16** | -83967 | -109596 to -58338 | Yes | **** | <0.0001 |
| **fish10 vs. fish17** | -23793 | -49421 to 1836 | No | ns | 0.1135 |
| **fish10 vs. fish18** | 16153 | -9475 to 41782 | No | ns | >0.9999 |
| **fish11 vs. fish12** | 32311 | 6683 to 57940 | Yes | ** | 0.0022 |
| **fish11 vs. fish13** | 5033 | -20596 to 30662 | No | ns | >0.9999 |
| **fish11 vs. fish14** | -2887 | -28516 to 22742 | No | ns | >0.9999 |
| **fish11 vs. fish15** | 61430 | 35801 to 87058 | Yes | **** | <0.0001 |
| **fish11 vs. fish16** | -14602 | -40231 to 11026 | No | ns | >0.9999 |
| **fish11 vs. fish17** | 45572 | 19943 to 71201 | Yes | **** | <0.0001 |
| **fish11 vs. fish18** | 85518 | 59889 to 111147 | Yes | **** | <0.0001 |
| **fish12 vs. fish13** | -27278 | -52907 to -1650 | Yes | * | 0.0236 |
| **fish12 vs. fish14** | -35198 | -60827 to -9570 | Yes | *** | 0.0006 |
| **fish12 vs. fish15** | 29118 | 3490 to 54747 | Yes | * | 0.0101 |
| **fish12 vs. fish16** | -46914 | -72542 to -21285 | Yes | **** | <0.0001 |
| **fish12 vs. fish17** | 13261 | -12368 to 38889 | No | ns | >0.9999 |
| **fish12 vs. fish18** | 53207 | 27578 to 78835 | Yes | **** | <0.0001 |
| **fish13 vs. fish14** | -7920 | -33549 to 17709 | No | ns | >0.9999 |
| **fish13 vs. fish15** | 56397 | 30768 to 82025 | Yes | **** | <0.0001 |
| **fish13 vs. fish16** | -19635 | -45264 to 5993 | No | ns | 0.6658 |
| **fish13 vs. fish17** | 40539 | 14910 to 66168 | Yes | **** | <0.0001 |
| **fish13 vs. fish18** | 80485 | 54856 to 106114 | Yes | **** | <0.0001 |
| **fish14 vs. fish15** | 64317 | 38688 to 89945 | Yes | **** | <0.0001 |
| **fish14 vs. fish16** | -11715 | -37344 to 13913 | No | ns | >0.9999 |
| **fish14 vs. fish17** | 48459 | 22830 to 74088 | Yes | **** | <0.0001 |
| **fish14 vs. fish18** | 88405 | 62776 to 114034 | Yes | **** | <0.0001 |
| **fish15 vs. fish16** | -76032 | -101661 to -50403 | Yes | **** | <0.0001 |
| **fish15 vs. fish17** | -15858 | -41486 to 9771 | No | ns | >0.9999 |
| **fish15 vs. fish18** | 24088 | -1540 to 49717 | No | ns | 0.0996 |
| **fish16 vs. fish17** | 60174 | 34546 to 85803 | Yes | **** | <0.0001 |
| **fish16 vs. fish18** | 100120 | 74492 to 125749 | Yes | **** | <0.0001 |
| **fish17 vs. fish18** | 39946 | 14317 to 65575 | Yes | **** | <0.0001 |
